# Optimal Stopping Ages for Colorectal Cancer Screening

**DOI:** 10.1001/jamanetworkopen.2024.51715

**Published:** 2024-12-19

**Authors:** Matthias Harlass, Ronit R. Dalmat, Jessica Chubak, Rosita van den Puttelaar, Natalia Udaltsova, Douglas A. Corley, Christopher D. Jensen, Nicholson Collier, Jonathan Ozik, Iris Lansdorp-Vogelaar, Reinier G.S. Meester

**Affiliations:** 1 Department of Public Health, Erasmus University Medical Center, Rotterdam, South Holland, the Netherlands; 2 Department of Global Health, University of Washington, Seattle; 3 Department of Epidemiology, University of Washington, Seattle; 4 Kaiser Permanente Washington Health Research Institute, Seattle; 5 Division of Research, Kaiser Permanente Northern California, Oakland; 6 Department of Gastroenterology, Kaiser Permanente San Francisco Medical Center, San Francisco, California; 7 Decision and Infrastructure Sciences, Argonne National Laboratory, Lemont, Illinois; 8 Consortium for Advanced Science and Engineering, The University of Chicago, Chicago, Illinois; 9 Health Economics and Outcomes Research, Freenome Holdings Inc, South San Francisco, California; 10 Stanford University School of Medicine, Stanford, California

## Abstract

**Importance:**

Prior studies have shown that the benefits, harms, and costs of colorectal cancer (CRC) screening at older ages are associated with a patient’s sex, health, and screening history. However, these studies were hypothetical exercises and not directly informed by data on CRC risk.

**Objective:**

To identify the optimal stopping ages for CRC screening by sex, comorbidity, and screening history from a cost-effectiveness perspective.

**Design, Setting, and Participants:**

This economic evaluation first validated the MISCAN-Colon (Microsimulation Screening Analysis–Colon) model against community-based CRC incidence and mortality rates for 2 subcohorts of the PRECISE (Optimizing Colorectal Cancer Screening Precision and Outcomes in Community-Based Populations) cohort. Subsequently, different CRC screening scenarios were simulated in older individuals. Cohorts of US adults aged 76 to 90 years varied by sex and comorbidity status (none, low, moderate, or severe). Statistical and sensitivity analyses were performed from March 2023 to May 2024.

**Exposures:**

CRC screening histories including fecal immunochemical test (FIT) or colonoscopy, such as a negative colonoscopy result from 10, 15, 20, 25, or 30 years before the index age; 1 to 5 negative FIT results within 5 years of the index age, with different patterns of recency; or a combination of negative colonoscopy and negative FIT results.

**Main Outcomes and Measures:**

The main outcomes included estimated lifetime clinical outcomes, incremental costs, and quality-adjusted life-years gained (QALYG) associated with 1 additional FIT or colonoscopy. Optimal stopping age for screening, defined as the oldest age for which the incremental cost-effectiveness ratio was still below the willingness-to-pay threshold of $100 000 per QALYG, was evaluated.

**Results:**

The first of the 2 PRECISE subcohorts used in validating the simulation model included 25 974 adults (15 060 females [58.0%]; 54.7% aged 76 to 80 years) with a negative colonoscopy result 10 years before the index date. The second subcohort consisted of 118 269 adults (67 058 females [56.7%]; 90.5% aged 76 to 80 years) with a negative FIT result 1 year before the index date. Older age, male sex, higher comorbidity levels, and recent CRC screenings were associated with reduced incremental benefit and cost-effectiveness of additional screening. For the reference cohort of 76-year-old females without comorbidities and a negative colonoscopy result 10 years before the index age, 1 additional colonoscopy cost $38 226 per QALYG. For cohorts with otherwise equivalent characteristics, associated costs increased to $1 689 945 per QALYG for females at age 90 years without comorbidities and a negative colonoscopy results 10 years before the index age, $51 604 per QALYG for males at age 76 years without comorbidities and a negative colonoscopy result 10 years before the index age, and $108 480 per QALYG for females at age 76 years with severe comorbidities and a negative colonoscopy result 10 years before the index age and decreased to $16 870 per QALYG for females without comorbidities and a negative colonoscopy result 30 years before the index age. The optimal stopping ages across different cohorts ranged from younger than 76 to 86 years for colonoscopy and younger than 76 to 88 years for FIT.

**Conclusions and Relevance:**

In this economic evaluation, age, sex, screening history, comorbidity, and future screening modality were associated with the clinical outcomes, cost-effectiveness, and optimal stopping age for CRC screening. These results can inform guideline development and patient-directed informed decision-making.

## Key Points

**Question:**

What is the optimal stopping age for colorectal cancer (CRC) screening in the US from a cost-effectiveness perspective, considering an individual’s sex, comorbidity status, and screening history?

**Findings:**

In this economic evaluation using simulation data validated with community-based outcomes data, the optimal stopping ages ranged from younger than 76 to 86 years for colonoscopy and from younger than 76 to 88 years for fecal immunochemical testing, depending on the individual’s sex, comorbidity status, and screening history.

**Meaning:**

These findings can inform guideline development and patient-directed informed decision-making.

## Teaser

This economic evaluation uses community-based data to estimate outcomes, costs, and life-years gained associated with additional fecal immunochemical testing or colonoscopy for colorectal cancer.

## Introduction

For adults between the ages of 76 and 85 years, the US Preventive Services Task Force (USPSTF) advises shared decision-making and selective colorectal cancer (CRC) screening based on the person’s overall health, screening history, and personal preferences.^1^ The burden and benefits of CRC screening depend on the individual’s overall life expectancy and CRC risk.

Older age, comorbidity, and male sex are associated with reduced life expectancy, potentially offsetting the benefits of cancer screening.^2,3^ Sex and absence of prior screening are among the risk factors for developing CRC. Males have higher CRC incidence and mortality rates than females.^4^ Screening is associated with lower CRC risk, and individuals with up-to-date screening may benefit less from additional tests after age 75 years.^5^ Differences in these patient characteristics provide promising targets for personalized screening approaches. Previous studies investigated the optimal CRC stopping ages in a limited number of scenarios or alternative settings^6,7^ and did not validate their findings against community-based data in the US. Therefore, little evidence is available to guide decision-making regarding the optimal stopping age and screening modality moving forward for more complex combinations of patient characteristics.

In this economic evaluation, we aimed to identify the optimal stopping ages for CRC screening by sex, comorbidity, and screening history from a cost-effectiveness perspective. Specifically, we validated a microsimulation model’s estimates of CRC incidence and mortality after age 75 years against community-based data from Kaiser Permanente and ascertained the optimal stopping age for CRC screening using fecal immunochemical test (FIT) or colonoscopy by sex, comorbidity status, and screening history. The results can help patients and clinicians evaluate if and by which strategy screening may be appropriate after age 75 years.

## Methods

In this economic evaluation, we validated the Microsimulation Screening Analysis–Colon (MISCAN-Colon) model distinguishing age, sex, and comorbidity status against community-based data on CRC incidence and mortality from 3 integrated health care systems in the US (Kaiser Permanente in Northern California, Southern California, and Washington). Subsequently, we simulated the estimated benefits, harms, and costs of CRC screening between ages 76 and 90 years by sex, comorbidity, and prior screening. We estimated the optimal stopping ages for screening based on the incremental costs and quality-adjusted life-years gained (QALYG) associated with 1 additional FIT or colonoscopy. We calculated the outcomes of screening each simulated cohort from age 76 years until the estimated optimal stopping age. The institutional review board for each participating institution that contributed data approved this study and waived the informed consent requirement in accordance with the Common Rule. We followed the Consolidated Health Economic Evaluation Reporting Standards (CHEERS) reporting guideline.^8^

### Validation Data and Microsimulation Model

The validation data came from 3 distinct integrated health care systems, each with different patient populations, demographic characteristics, screening practices, and regional characteristics, contributing to the Optimizing Colorectal Cancer Screening Precision and Outcomes in Community-Based Populations (PRECISE) cohort.^9,10^ These populations include almost 1 of every 30 people in the US and approximate the demographic characteristics of the health care system’s region compared with Census data.^11^ The results of a retrospective study on cumulative CRC incidence and CRC mortality among older adults in the PRECISE cohort have been previously published.^10,12^ We used CRC incidence and CRC mortality rates for 2 subcohorts (from the PRECISE cohort) at 2, 5, and 8 years of follow-up from when these patients would have next been eligible for screening, stratified by age (76-80 or 81-85 years), sex (male or female), and comorbidity status (Charlson Comorbidity Index [CCI] score 1 year before entering the validation cohort: 0, 1, 2, 3, 4, or ≥5, with the highest score indicating severe comorbidity). Associations of race and ethnicity with estimated outcomes were not analyzed, although race and ethnicity data are reported to provide information about the validation cohorts.

The MISCAN-Colon model was developed by the Department of Public Health from Erasmus University Medical Center in Rotterdam, the Netherlands. It is part of the National Cancer Institute–funded Cancer Intervention and Surveillance Modeling Network and has previously informed screening recommendations by the USPSTF.^13^ An extensive description of the MISCAN-Colon model structure has been published elsewhere,^14,15^ and a brief description is provided in the eAppendix in Supplement 1.

### Study Population

We modeled 6480 US cohorts for all permutations of index age (76-90 years), sex (male or female), comorbidity status (none, low, moderate, or severe), and screening history (none, FIT, colonoscopy, or combination of both). For each cohort, we simulated approximately 250 million individuals without prior cancer diagnosis or positive test results in their screening history. We opted for the large population size to be able to detect minor differences between near-identical cohorts with few CRC events.

### Sex, Comorbidity Status, and Screening History

We used sex-specific CRC background risk parameters and sex- and stage-specific survival times after CRC diagnosis similar to previous modeling analyses for the USPSTF.^13^ We applied incidence rate ratios of 1.28 for males and 1.19 for females vs the 1975 to 1979 CRC incidence data from the Surveillance, Epidemiology, and End Results Program used to inform the model’s background risk to account for patterns of increasing incidence among individuals aged 20 to 44 years.

We used age-, sex-, and comorbidity-specific life tables for the US. The conditions included in each comorbidity category (none, low, moderate, or severe) have been described previously.^2,3^ We assumed that comorbidities affect only the probability of death from noncancer causes and not cancer background risk, progression, or survival.

Simulated screening histories included a single negative colonoscopy result from 10, 15, 20, 25, or 30 years before the index age; 1 to 5 negative FIT results within 5 years of the index age, with different patterns of recency; or a combination of negative colonoscopy and negative FIT results. All simulated screening histories, test performance, and polyp surveillance assumptions are presented in eTables 1 to 3 in Supplement 1. We assumed 100% adherence to all screening and surveillance tests.

### Statistical Analysis

Simulations and postprocessing were performed from March 2023 to May 2024 using Python 3.9 (Python Software Foundation). Figures and the complementary web application were created using R, version 4.2.2 (R Foundation for Statistical Computing).

#### Costs and Utilities

All costs were calculated from a modified societal perspective, which included direct medical and patient time costs but excluded direct nonmedical and broader societal spillover implications (eTables 4 and 5 in Supplement 1). The costs for FIT, screening-related complications, and cancer care were updated from Peterse et al^16^ to 2020 US dollars using the Consumer Price Index.^17^ Colonoscopy costs were calculated using 2020 Centers for Medicare & Medicaid Services data.

The assumed loss in quality of life associated with CRC care, screening, and complications is described in eTables 6 and 7 in Supplement 1. Utilities and costs were discounted at an annual rate of 3%.

#### Model Validation

To validate the model, we simulated cohorts who varied in sex and comorbidity status with either a negative colonoscopy result 10 years before or a negative FIT result 1 year before the index age between 76 and 85 years and different numbers of FIT tests in the 5 years before the index age to reflect heterogeneity in the observed data. The comorbidity statuses in the simulation model (none, low, moderate, and severe) were based on the comorbidity-specific life-expectancy estimates by Cho et al.^2^ The comorbidity status in the community-based data was based on the CCI scores and was mapped to the categories used in the simulation. Individuals with a CCI score of 0 were categorized as having none, 1 or 2 as having low comorbidity, 3 or 4 as having moderate comorbidity, and 5 or higher as having severe comorbidity. For each simulated cohort, we then estimated the CRC incidence and CRC mortality rates at 2, 5, and 8 years after the index age. We compared the model-estimated rates with observed rates by age, sex, comorbidity status, and number of prior FITs.

#### Outcomes

For each of the 6480 modeled cohorts, we evaluated 3 scenarios: (1) 1 additional FIT, (2) 1 additional colonoscopy, and (3) no further screening. We calculated the following outcomes per 1000 individuals for all cohorts: CRC cases, CRC deaths, lifetime colonoscopies, complications, life-years, quality-adjusted life-years, and costs. We then compared the outcomes of scenarios with additional FIT or colonoscopy against the corresponding scenario without further screening. We defined the optimal stopping age as the oldest age at which the incremental cost-effectiveness ratio (ICER) of additional FIT or colonoscopy remained below the most commonly used willingness-to-pay threshold in the US literature of $100 000 per QALYG. Alternative thresholds can be evaluated using the complementary web application we developed.^18^

#### Sensitivity Analyses

We conducted multiple sensitivity analyses. First, we included a sensitivity analysis assuming no surveillance after positive test results to assess the implications of postpolypectomy surveillance. Second, we assumed that colonoscopy complication rates increased with comorbidity status. A detailed description of the methods for the sensitivity analyses is provided in the eAppendix in Supplement 1.

## Results

Two subcohorts from the PRECISE cohort were used to validate the MISCAN-Colon model against community-based CRC incidence and mortality. The first subcohort included 25 974 adults with a negative colonoscopy result 10 years before the index date. This group was composed of 15 060 females (58.0%) and 10 914 males (42.0%), of whom 54.7% were aged 76 to 80 years and 0.3% identified as American Indian or Alaska Native, 11.3% as Asian, 9.1% as Black, 13.4% as Hispanic, 0.4% as Native Hawaiian and Other Pacific Islander, and 66.2% as White individuals; 13.9% had no race information. The group had a cumulative 8-year CRC incidence of 1.29%. The second subcohort consisted of 118 269 adults with a negative FIT result 1 year before the index date. This group consisted of 67 106 females (56.7%) and 51 161 males (43.3%), of whom 90.5% were aged 76 to 80 years and 0.4% identified as American Indian or Alaska Native, 11.9% as Asian, 7.6% as Black, 15.5% as Hispanic, 0.5% as Native Hawaiian and Other Pacific Islander, and 63.8% as White individuals; 1.1% were missing race information. The group had a cumulative 8-year CRC incidence of 1.21%.

**Figure 1.**
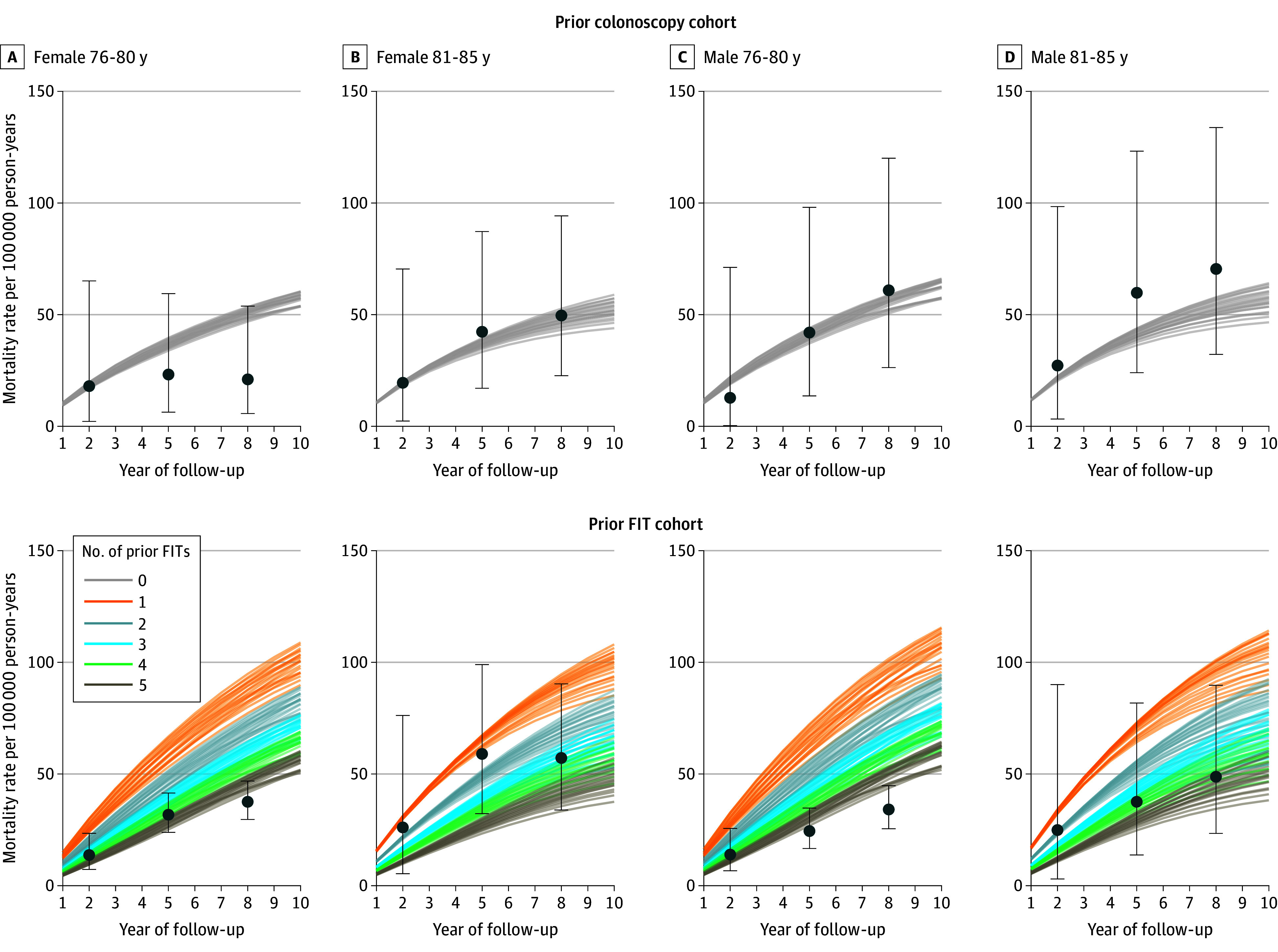
Sex- and Age-Specific Colorectal Cancer Mortality Rates for Cohorts With a Negative Colonoscopy Result 10 Years Before Index Age and Cohorts With ≥1 Negative Fecal Immunochemical Test (FIT) Result Within 5 Years Before Index Age The points indicate the observed mortality, and error bars indicate 95% CIs. The lines represent the simulated mortality for each index age (76-85 years) over 10 years of follow-up, stratified by number of FITs over the past 5 years.

The model-estimated CRC mortality rates ranged from 17.1 to 19.8 per 100 000 person-years at 2 years, from 33.9 to 39.6 per 100 000 person-years at 5 years, and from 46.9 to 53.5 per 100 000 person-years at 8 years of follow-up in females aged 76 to 80 years with a colonoscopy 10 years before the index age (Figure 1). In males, the estimated mortality rates were slightly higher. The estimated rates for individuals aged 81 to 85 years were comparable to the younger age group and followed the same sex-specific pattern. For the prior colonoscopy cohorts, all model-estimated rates were within the CIs of rates observed in the first PRECISE subcohort.

The model estimated that CRC incidence rates ranged from 133.2 to 147.9 per 100 000 person-years at 2 years, from 156.6 to 168.1 per 100 000 person-years at 5 years, and from 175.7 to 184.4 per 100 000 person-years at 8 years for female cohorts aged 76 to 80 years with a negative colonoscopy result 10 years before the index age (eFigure 1 in Supplement 1). The estimated incidence for younger cohorts was marginally higher than for older cohorts and slightly lower in females than in males (eFigure 1 in Supplement 1).

Estimated CRC incidence and mortality rates varied more for the prior FIT cohorts and may have overestimated mortality in the younger male cohorts. However, the model closely matched the older cohorts’ incidence and mortality rates. The incidence and mortality rates by comorbidity status followed similar patterns (eFigures 6-7 in Supplement 1). A sensitivity analysis to assess the implications of screening more than 5 years prior is described in the eAppendix and eFigures 2-5 in Supplement 1.

### Lifetime Clinical Outcomes and Resource Impact

The clinical outcomes for a reference cohort (females without comorbidities and a negative colonoscopy result 10 years before the index age) are presented in Table 1 and Table 2, while results for all cohorts are available in eTable 8 in Supplement 2. We observed the highest estimated number of life-years gained (eg, 101.11 per 1000 individuals with no screening history) and CRC deaths averted (eg, 17.52 per 1000 individuals with no screening history) at age 76 years and a sharp reduction in these benefits for older ages.

**Table 1.** Clinical Outcomes of 1 Additional Colonoscopy

Cohort characteristic^a^	Index age, y	Outcomes per 1000 individuals, No.				
		Incremental colonoscopies	Incremental complications	CRC cases averted	CRC deaths averted	Life-years gained
Sex						
Female	76	1206.49	31.20	2.85	6.38	36.35
	81	1057.98	34.63	−0.33	4.33	20.47
	86	1039.74	45.23	−4.13	2.32	8.07
	90	1044.27	56.74	−4.82	1.03	2.66
Male	76	1209.48	31.75	0.42	5.80	30.08
	81	1060.12	35.30	−2.52	3.99	17.34
	86	1040.08	45.96	−6.20	2.08	6.78
	90	1044.26	57.54	−6.11	0.97	2.50
Comorbidity						
None	76	1206.49	31.20	2.85	6.38	36.35
	81	1057.98	34.63	−0.33	4.33	20.47
	86	1039.74	45.23	−4.13	2.32	8.07
	90	1044.27	56.74	−4.82	1.03	2.66
Low	76	1191.27	30.61	0.40	5.19	28.07
	81	1058.83	34.68	−1.93	3.67	16.70
	86	1040.35	45.27	−4.75	2.11	7.09
	90	1044.57	56.76	−5.12	0.96	2.43
Moderate	76	1184.23	30.33	−0.84	4.59	23.88
	81	1059.26	34.70	−3.19	3.17	13.26
	86	1041.55	45.34	−5.94	1.72	5.31
	90	1045.20	56.80	−5.74	0.78	1.84
Severe	76	1153.28	29.11	−3.31	3.48	17.05
	81	1058.57	34.67	−6.07	2.18	8.55
	86	1043.59	45.45	−7.98	1.14	3.31
	90	1046.25	56.87	−6.79	0.54	1.19
Screening history						
None	76	1470.28	46.66	−2.05	17.52	101.11
	81	1172.35	46.50	−14.15	13.03	62.52
	86	1011.05	52.61	−29.11	8.19	29.13
	90	1019.76	67.41	−39.45	4.94	13.11
Colonoscopy 10 y before index age	76	1206.49	31.20	2.85	6.38	36.35
	81	1057.98	34.63	−0.33	4.33	20.47
	86	1039.74	45.23	−4.13	2.32	8.07
	90	1044.27	56.74	−4.82	1.03	2.66
5 Recent FITs	76	1347.50	39.58	12.76	8.48	44.89
	81	1113.68	40.78	9.44	5.44	23.78
	86	1039.27	50.26	4.12	2.77	9.07
	90	1043.45	63.75	0.61	1.36	3.33
Colonoscopy 20 y before index age plus 5 recent FITs	76	1237.32	33.10	9.17	5.95	31.83
	81	1064.27	35.57	6.49	3.67	16.14
	86	1043.11	46.20	2.74	1.78	5.75
	90	1046.14	58.08	0.47	0.80	2.07

Abbreviations: CRC, colorectal cancer; FIT, fecal immunochemical test.

^a^ Results are presented for cohorts matching a reference cohort (females without comorbidities and colonoscopy 10 years before index age) except for the single parameter that varied in each section (sex, comorbidity, or screening history). This approach allowed for isolated assessment of each factor’s role in outcomes. Clinical outcomes and cost-effectiveness estimates for other cohorts are provided in eTable 8 in Supplement 2.

**Table 2.** Clinical Outcomes of 1 Additional FIT

Cohort characteristic^a^	Index age, y	Outcomes per 1000 individuals, No.				
		Incremental colonoscopies	Incremental complications	CRC cases averted	CRC deaths averted	Life-years gained
Sex						
Female	76	61.59	1.77	−4.43	1.67	11.03
	81	51.10	1.83	−5.69	1.35	7.23
	86	47.92	2.29	−6.19	0.83	3.18
	90	47.61	2.85	−5.11	0.40	1.20
Male	76	63.38	1.86	−5.46	1.60	9.45
	81	52.98	1.94	−6.90	1.29	6.21
	86	49.60	2.41	−7.40	0.77	2.77
	90	49.05	2.99	−5.97	0.39	1.05
Comorbidity						
None	76	61.59	1.77	−4.43	1.67	11.03
	81	51.10	1.83	−5.69	1.35	7.23
	86	47.92	2.29	−6.19	0.83	3.18
	90	47.61	2.85	−5.11	0.40	1.20
Low	76	60.71	1.74	−4.95	1.42	8.57
	81	51.37	1.85	−6.09	1.17	6.03
	86	48.10	2.30	−6.38	0.76	2.81
	90	47.72	2.86	−5.22	0.37	1.10
Moderate	76	60.20	1.72	−5.20	1.28	7.48
	81	51.57	1.86	−6.43	1.04	4.87
	86	48.49	2.32	−6.77	0.64	2.15
	90	47.93	2.87	−5.44	0.31	0.84
Severe	76	58.08	1.63	−5.90	1.01	5.31
	81	51.82	1.86	−7.31	0.74	3.14
	86	49.23	2.35	−7.49	0.44	1.36
	90	48.36	2.90	−5.84	0.22	0.62
Screening history						
None	76	166.15	6.11	−16.44	6.37	40.71
	81	131.81	6.08	−24.13	5.51	29.07
	86	99.86	6.08	−31.50	3.93	15.02
	90	106.82	8.26	−36.53	2.60	7.27
Colonoscopy 10 y before index age	76	62.69	1.88	−0.20	1.09	6.63
	81	49.78	1.84	−0.83	0.82	4.03
	86	45.78	2.23	−1.04	0.45	1.49
	90	45.77	2.80	−1.20	0.22	0.56
5 Recent FITs	76	61.59	1.77	−4.43	1.67	11.03
	81	51.10	1.83	−5.69	1.35	7.23
	86	47.92	2.29	−6.19	0.83	3.18
	90	47.61	2.85	−5.11	0.40	1.20
Colonoscopy 20 y before index age plus 5 recent FITs	76	95.14	3.26	0.14	1.77	10.23
	81	74.69	3.21	−0.82	1.38	6.68
	86	61.37	3.50	−1.48	0.78	2.58
	90	62.77	4.55	−2.03	0.43	1.14

Abbreviations: CRC, colorectal cancer; FIT, fecal immunochemical test.

^a^ Results are presented for cohorts matching a reference cohort (females without comorbidities and colonoscopy 10 years before index age) except for the single parameter that varied in each section (sex, comorbidity, or screening history). This approach allowed for isolated assessment of each factor’s role in outcomes. Clinical outcomes and cost-effectiveness estimates for other cohorts are provided in eTable 8 in Supplement 2.

Concurrently, the number of CRC cases averted was estimated to decrease with age, reaching negative values for most cohorts, indicating more frequent overdiagnosis at older ages. For instance, conducting an additional colonoscopy for 76-year-old females without comorbidities and a negative colonoscopy result from 10 years before the index age was associated with an estimated 2.85 CRC cases averted and 6.38 CRC deaths prevented as well as an estimated 36.35 life-years gained per 1000 individuals. The screening required 1206.49 additional colonoscopies, which was associated with 31.20 complications per 1000 individuals. In contrast, an additional colonoscopy at age 86 years for an equivalent cohort was associated with −4.13 CRC cases averted, 2.32 CRC deaths averted per 1000 individuals, and 8.07 life-years gained per 1000 individuals while requiring 1039.74 additional colonoscopies. Compared with colonoscopy, incremental FIT was associated with fewer deaths averted (1.67 vs 6.38 per 1000 individuals with colonoscopy), fewer life-years gained (11.03 vs 36.35 per 1000 individuals with colonoscopy), and more CRC cases diagnosed (–4.43 vs 2.85 CRC cases averted per 1000 individuals with colonoscopy). FIT was also associated with fewer estimated colonoscopies (61.59 vs 1206.49 per 1000 individuals with colonoscopy) and colonoscopy-related complications (1.77 vs 31.20 per 1000 individuals with colonoscopy) (Table 1 and Table 2).

Sex, comorbidity status, and screening history were associated with clinical outcomes and resource utilization. With additional screening, female cohorts had slightly more estimated life-years gained and CRC deaths averted than male cohorts. For example, at age 76 years, males without comorbidities had 6.3 fewer estimated life-years gained (30.08 vs 36.35) per 1000 individuals and 0.6 fewer estimated CRC deaths averted (5.80 vs 6.38) per 1000 individuals from 1 more colonoscopy than their female counterparts. The relative difference by sex diminished with increasing age.

Comorbidity status was associated with estimated lifetime clinical outcomes. For 76-year-old females with a colonoscopy conducted 10 years before the index age, the number of CRC deaths averted associated with an additional colonoscopy decreased from 6.38 to 5.19, 4.59, and 3.48 per 1000 individuals as comorbidity increased from none to low, moderate, and severe, respectively. Similarly, the estimated life-years gained associated with increasing comorbidity decreased from 36.35 to 28.07, 23.88, and 17.05 per 1000 individuals.

The estimated burden and benefits of screening after age 75 years also varied considerably by screening history, with screening-naive cohorts experiencing the largest estimated gain in life-years and averted CRC deaths. For females aged 76 years without prior screening, colonoscopy was associated with 101.11 life-years gained per 1000 individuals and 17.52 CRC deaths averted per 1000 individuals. In the same cohort, FIT was associated with substantially fewer incremental colonoscopies (166.15 per 1000 individuals) but also fewer estimated life-years gained (40.71 per 1000 individuals) and CRC deaths averted (6.37 per 1000 individuals) (Table 2).

### Optimal Stopping Ages

**Figure 2.**
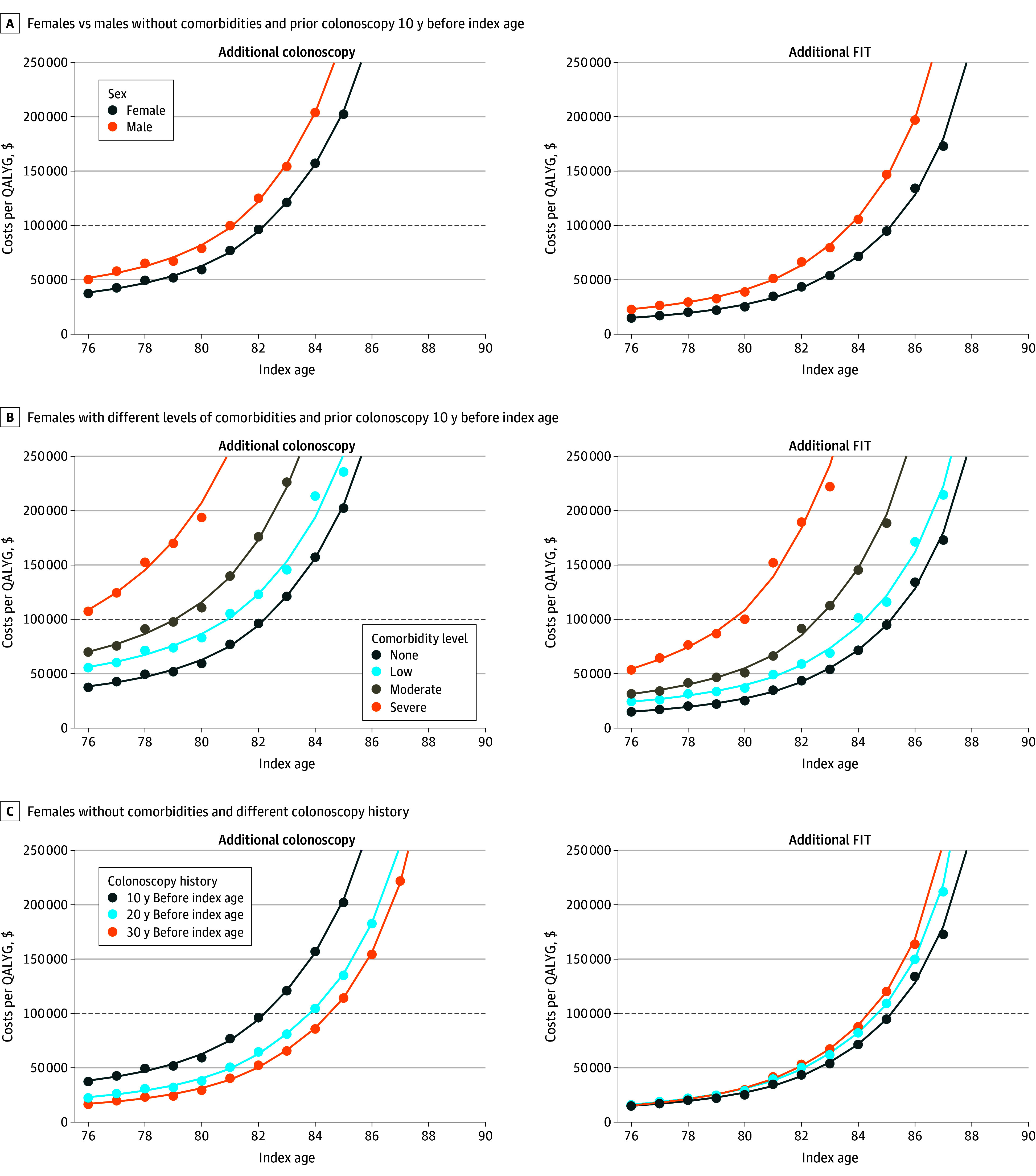
Costs per Quality-Adjusted Life-Year Gained (QALYG) for Selected Strategies Stratified by Sex, Comorbidity, and Screening History The points represent the estimated cost per QALYG with 1 additional colonoscopy and fecal immunochemical test (FIT). The colored lines indicate the smoothed incremental cost-effectiveness ratios calculated from smoothed costs and QALYGs. The dashed horizontal line represents the willingness-to-pay threshold of $100 000 per QALYG.

The estimated patterns in clinical outcomes by sex, comorbidity status, and screening history were associated with different cost-effectiveness ratios and stopping ages for additional screening. Screening after age 75 years was associated with more favorable ICERs for females, individuals with lower comorbidity levels, and individuals with less intensive and less recent prior screening (Figure 2). For the reference cohort of females aged 76 years without comorbidities and a negative colonoscopy result 10 years before the index age, 1 additional colonoscopy cost $38 226 per QALYG. For cohorts with otherwise equivalent characteristics, associated costs increased to $1 689 945 per QALYG for females at age 90 years without comorbidities and a negative colonoscopy results 10 years before the index age, $51 604 per QALYG for males at age 76 years without comorbidities and a negative colonoscopy result 10 years before the index age, and $108 480 per QALYG for females at age 76 years with severe comorbidities and a negative colonoscopy result 10 years before the index age and decreased to $16 870 per QALYG for females without comorbidities and a negative colonoscopy result 30 years before the index age. FIT was associated with lower estimated ICERs compared with colonoscopy for all screening histories except for screening-naive cohorts. The estimated optimal stopping ages varied accordingly and ranged from younger than 76 to 86 years for colonoscopy and younger than 76 to 88 years for FIT. Data on the clinical outcomes and resources associated with the continuation of FIT from age 76 years until the optimal stopping age are provided in eFigure 15 in Supplement 1 and eTable 9 in Supplement 2. Moreover, the optimal stopping ages at other willingness-to-pay thresholds can be evaluated using our web application.^18^

**Figure 3.**
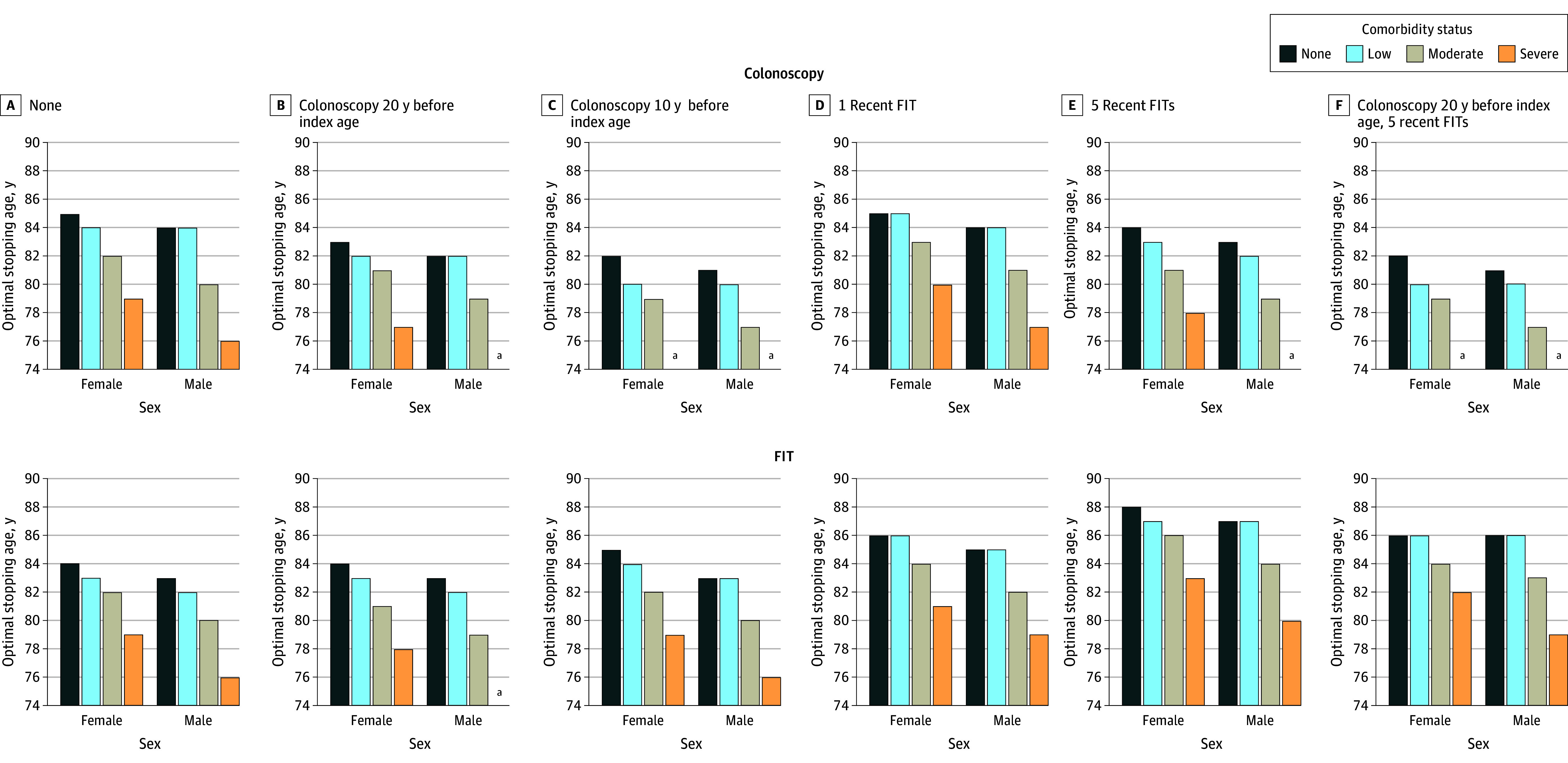
Optimal Colorectal Cancer Screening Stopping Age by Modality for Selected Screening Histories The optimal stopping age is defined as the oldest age at which 1 additional screening is still cost-effective. Figures for other screening histories are provided in eFigures 8-14 in Supplement 1. FIT indicates fecal immunochemical test. ^a^Screening after age 75 years is not cost-effective.

For colonoscopy, the optimal stopping ages in cohorts without comorbidities were from 81 to 86 years for females and from 80 to 84 years for males depending on screening history. The optimal stopping ages for FIT across all screening histories were higher for those without comorbidities: between 84 and 88 years for females and between 82 and 87 years for males (Figure 3; eFigures 8-14 in Supplement 1).

Comorbidity status was associated with the estimated cost-effectiveness and optimal stopping ages for screening. Optimal stopping ages for both sexes by comorbidity status and screening history are presented in Figure 3 and eFigures 8-14 in Supplement 1. The optimal ages to stop FIT for females with a negative colonoscopy result 10 years before the index age were 85 years for none, 84 years for low, 82 years for moderate, and 79 years for severe comorbidity status. Screening these cohorts with colonoscopy was only cost-effective for females with no, low, or moderate comorbidities and not beyond age 82 years. Across all screening histories, the optimal colonoscopy stopping age for females ranged from 81 to 86 years for none, 79 to 85 years for low, 77 to 83 years for moderate, and under 76 to 80 years for severe comorbidity status. The optimal ages to stop FIT followed the same decreasing pattern but were older.

The optimal stopping ages for colonoscopy in cohorts without comorbidities were 82 years, 83 years, and 84 years for females with a negative colonoscopy result from 10, 20, and 30 years before the index age, respectively. Overall, the optimal stopping age for female cohorts without comorbidity varied by screening history from 81 to 86 years for colonoscopy and 84 to 88 years for FIT.

The estimated cost-effectiveness and optimal stopping ages did not change meaningfully when assuming no surveillance after a colonoscopy in which adenomas were detected (eFigure 16 in Supplement 1). Patterns of estimated ICERs across cohorts were slightly lower at age 76 years and rapidly converged with the estimates assuming surveillance. Without postpolypectomy surveillance, the optimal stopping ages were, on average, 80.05 years (0.09 years older) for colonoscopy and 83.14 years (0.04 years older) for FIT.

Using comorbidity status–specific colonoscopy complication rates only had a minor implication for estimating clinical outcomes and optimal stopping ages. On average, the optimal stopping ages for colonoscopy increased to 82.73 years (by 0.12 years) for cohorts without and to 81.71 years (by 0.02 years) for cohorts with low comorbidities, whereas they decreased to 79.12 years (by 0.31 years) for cohorts with moderate and to 75.72 years (by 0.38 years) for cohorts with severe comorbidities. In comparison, the changes in optimal stopping ages for FIT were smaller.

## Discussion

This study suggests that the cost-effectiveness of CRC screening after age 75 years varies considerably by a person’s sex, comorbidity status, and screening history. The MISCAN-Colon model’s optimal stopping ages varied by more than 10 years between simulated cohorts. Overall, the estimated cost-effectiveness was less favorable for males than females, by older age, higher comorbidity status, and more intensive recent screening. FIT was cost-effective into later ages than colonoscopy, indicating that the optimal stopping age depends on the test modality. For example, although colonoscopy was not cost-effective after age 75 years in females with a negative colonoscopy result from 10 years before the index age and severe comorbidity status, FIT remained cost-effective until age 79 years.

The benefits of CRC screening depend on life expectancy and cancer risk. As expected, the results suggest older optimal stopping ages for cohorts with lower comorbidity statuses because they have a longer life expectancy. In contrast, prior screening with negative results was associated with lower CRC risk and less favorable cost-effectiveness of additional screening. Despite a lower CRC risk for females than males, the results showed that CRC screening at older ages is more cost-effective in females than males. This finding may seem counterintuitive at first given that for screening-naive populations, screening is more cost-effective in males.^13^ However, with some prior screening, the CRC risk difference is smaller, and the longer life expectancy becomes more important. Past studies have also suggested that screening benefits by sex converge with more recent and intensive prior screening and may become larger for females than males at older ages.^6,19^ Results of the present study showed a steep increase in cost per QALYG with age for all scenarios. In addition to the expected decrease in incremental QALYG with age, the cost increase can be explained by lower overall treatment benefits, less life expectancy gains, and more frequent complications at older ages. We also observed that at older ages, additional FIT had more favorable cost-effectiveness in cohorts with more recent prior screening, while the opposite was true for additional colonoscopy. This pattern surfaces as age increases and may be attributable to the larger relative increase in CRC overdiagnosis and a greater relative decrease in QALYG with FIT for cohorts with less recent prior screening.

This study expands findings from previous modeling analyses of optimal stopping ages for CRC screening of individuals older than 75 years with few comorbidities and limited prior screening.^3,6,7,15^ To our knowledge, the current study is the first to use a model validated against observed US community-based data to assess potential, evidence-based stopping ages for CRC screening. This analysis extends previous research by evaluating the cost-effectiveness of either additional FIT or colonoscopy while simultaneously considering the implications of sex, comorbidities, and prior screening. Moreover, we also considered hybrid screening histories. In practice, clinicians encounter a wide variety of prior screening modalities and adherence patterns, which may question the appropriateness of uniformly applying the current recommendations to all patients. Recommendations on hybrid screening strategies may also have gained relevance given that some patients switched from colonoscopy to FIT during the COVID-19 pandemic to limit in-person medical procedures.^20,21^ This study directly addressed the complexity of patient characteristics found routinely in clinical practice and may inform guideline development and patient-directed informed decision-making.

High degrees of heterogeneity in patient characteristics and difficulties in estimating competing mortality can make individualized decision-making about screening at older ages difficult. Therefore, we developed a web application as a decision aid that visualizes the estimated lifetime clinical outcomes, resource impact, cost-effectiveness, and optimal stopping age based on the entered current patient characteristics (age, sex, comorbidity status, and screening history). For example, if a male patient aged 76 years presents without comorbidities and with a negative colonoscopy result from 10 years ago, the web application would indicate that either 1 final colonoscopy or continuing FIT may have favorable clinical outcomes. However, if the patient returns the next year with severe comorbidities, a clinician could reevaluate the outcomes and advise to stop screening. The results of this study and the web tool can inform guideline development and patient-directed decision-making. However, the harm-benefit trade-off to patients using this information in the clinical setting warrants further investigation.

### Limitations

This study has several limitations. First, the model validation against CRC data from the PRECISE cohort showed higher estimated incidence and mortality rates than the observed rates for the younger cohorts with a negative FIT result from 1 year before the index age. Therefore, the results may overestimate cost-effectiveness for cohorts with FIT-only screening histories. We had no information on screening history in the validation cohorts beyond the most recent prior screening test prior to PRECISE cohort entry. Individuals in the PRECISE cohort who underwent additional screening prior to cohort enrollment would likely have a lower risk of CRC and CRC mortality. Thus, unobserved prior screening may explain discrepancies between simulated and observed incidence and mortality rates, and the sensitivity analysis of extensive prior screening supports this hypothesis. Second, we assumed 100% adherence to all tests. Therefore, we reported outcomes for individuals who were guideline adherent after a positive FIT result or detected polyps. However, we evaluated scenarios without surveillance, which did not materially affect the conclusions. Moreover, many of the simulated screening histories mirror scenarios of suboptimal adherence and give insight into the expected outcomes with lower screening uptake. For example, the simulated screening history of 3 negative FIT results from within 5 years before the index age may reflect reduced adherence to annual FIT. Similarly, a negative colonoscopy result from 15 or 20 years before the index age may reflect a missed colonoscopy under decennial colonoscopy screening. Third, we assumed that the comorbidity status affected only other-cause mortality and not cancer risk or survival. Comorbidities such as diabetes and cardiovascular diseases may not directly increase CRC risk or survival but can be associated with risk factors such as obesity.^22,23^ However, we evaluated the implications of increasing the colonoscopy complication rates for more severe comorbidity status and did not find a substantial change in clinical outcomes or optimal stopping ages.

## Conclusions

Findings from this economic evaluation using community-based data on CRC risk suggest that the clinical outcomes, cost-effectiveness, and optimal stopping age for CRC screening are associated with sex, comorbidity, prior screening, and future screening modality. Therefore, personalizing CRC screening based on these factors after age 75 years may play a role in the improvement of screening efficiency and reduction of potential harms.

## Notes

Supplement 1. **eAppendix.** Model Description, Model Validation Results, and Sensitivity Analyses **eTable 1**. Simulated Screening Histories Relative to Index Age **eTable 2.** Test Characteristics and Complications Assumptions **eTable 3.** Post-Colonoscopy Surveillance Assumptions **eTable 4.** Societal Costs of Screening, Follow-Up, Surveillance Tests, and Complications (2020 USD) **eTable 5.** Costs of Cancer Care (2020 USD) **eTable 6.** Utility Losses of Screening, Follow-Up, Surveillance Tests, and Complications **eTable 7.** Utility Losses of Cancer Care **eFigure 1**. Sex- and Age-Specific CRC Incidence Rates for Cohorts With a Negative Colonoscopy 10 Years Prior and Cohorts With ≥1 Negative FIT Prior **eFigure 2**. Sex- and Age-Specific CRC Incidence Rates by Number of Negative FIT Prior **eFigure 3**. Sex- and Age-Specific CRC-Mortality Rates by Number of Negative FIT Prior **eFigure 4**. Sex- and Age-Specific CRC Incidence Rates for Cohorts With ≥1 Negative FIT Prior and Cohorts With a Negative Colonoscopy + FIT (Hybrid) **eFigure 5**. Sex- and Age-Specific CRC-Mortality Rates for Cohorts With ≥1 Negative FIT Prior and Cohorts With a Negative Colonoscopy + FIT (Hybrid) **eFigure 6**. Comorbidity-Specific CRC Incidence Rates for Cohorts With a Negative Colonoscopy 10 Years Prior and Cohorts With ≥1 Negative FIT Prior **eFigure 7**. Comorbidity-Specific CRC-Mortality Rates for Cohorts With a Negative Colonoscopy 10 Years Prior and Cohorts With ≥1 Negative FIT Prior **eFigure 8**. Optimal CRC Screening Stopping Age by Modality for Screening Histories, Including No Screening or Only a Single Colonoscopy **eFigure 9**. Optimal CRC Screening Stopping Age by Modality for Screening Histories Only Including FITs **eFigure 10**. Optimal CRC Screening Stopping Age by Modality for Screening Histories, Including a Single Colonoscopy 10 Years Prior and 0-5 FIT **eFigure 11**. Optimal CRC Screening Stopping Age by Modality for Screening Histories, Including a Single Colonoscopy 15 Years Prior and 0-5 FIT **eFigure 12**. Optimal CRC Screening Stopping Age by Modality for Screening Histories, Including a Single Colonoscopy 20 Years Prior and 0-5 FIT **eFigure 13**. Optimal CRC Screening Stopping Age by Modality for Screening Histories, Including a Single Colonoscopy 25 Years Prior and 0-5 FIT **eFigure 14**. Optimal CRC Screening Stopping Age by Modality for Screening Histories, Including a Single Colonoscopy 30 Years Prior and 0-5 FIT **eFigure 15.** Estimated Clinical Outcomes of Continuing Annual FIT Screening for Cohorts From Age 76 Until the Estimated Stopping Age **eFigure 16.** Costs per QALY Gained (QALYG) for Selected Strategies Stratified by Post-Colonoscopy Surveillance Assumptions
eReferences

Supplement 2. **eTable 8.** Clinical Outcomes and Cost-Effectiveness Outcomes for All Cohorts **eTable 9.** Clinical Outcomes of FIT Screening From Age 76 Until the Optimal Stopping Age

Supplement 3.
Data Sharing Statement
