# Supplementary material for: Optimal Stopping Ages for Colorectal Cancer Screening
Source: JAMA Netw Open. 2024 Dec 19;7(12):e2451715. doi: 10.1001/jamanetworkopen.2024.51715 (PMC11659906; doi:10.1001/jamanetworkopen.2024.51715)
Supplement: Supplement 1. — eAppendix. Model Description, Model Validation Results, and Sensitivity Analyses eTable 1. Simulated Screening Histories Relative to Index Age eTable 2. Test Characteristics and Complications Assumptions eTable 3. Post-Colonoscopy Surveillance Assumptions eTable 4. Societal Costs of Screening, Follow-Up, Surveillance Tests, and Complications (2020 USD) eTable 5. Costs of Cancer Care (2020 USD) eTable 6. Utility Losses of Screening, Follow-Up, Surveillance Tests, and Complications eTable 7. Utility Losses of Cancer Care eFigure 1. Sex- and Age-Specific CRC Incidence Rates for Cohorts With a Negative Colonoscopy 10 Years Prior and Cohorts With ≥1 Negative FIT Prior eFigure 2. Sex- and Age-Specific CRC Incidence Rates by Number of Negative FIT Prior eFigure 3. Sex- and Age-Specific CRC-Mortality Rates by Number of Negative FIT Prior eFigure 4. Sex- and Age-Specific CRC Incidence Rates for Cohorts With ≥1 Negative FIT Prior and Cohorts With a Negative Colonoscopy + FIT (Hybrid) eFigure 5. Sex- and Age-Specific CRC-Mortality Rates for Cohorts With ≥1 Negative FIT Prior and Cohorts With a Negative Colonoscopy + FIT (Hybrid) eFigure 6. Comorbidity-Specific CRC Incidence Rates for Cohorts With a Negative Colonoscopy 10 Years Prior and Cohorts With ≥1 Negative FIT Prior eFigure 7. Comorbidity-Specific CRC-Mortality Rates for Cohorts With a Negative Colonoscopy 10 Years Prior and Cohorts With ≥1 Negative FIT Prior eFigure 8. Optimal CRC Screening Stopping Age by Modality for Screening Histories, Including No Screening or Only a Single Colonoscopy eFigure 9. Optimal CRC Screening Stopping Age by Modality for Screening Histories Only Including FITs eFigure 10. Optimal CRC Screening Stopping Age by Modality for Screening Histories, Including a Single Colonoscopy 10 Years Prior and 0-5 FIT eFigure 11. Optimal CRC Screening Stopping Age by Modality for Screening Histories, Including a Single Colonoscopy 15 Years Prior and 0-5 FIT eFigure 12. Optimal CRC Screening Stopping Age by M [file jamanetwopen-e2451715-s001.pdf]

Supplemental Online Content

Harlass M, Dalmat R, Chubak J, et al. Optimal stopping ages for colorectal cancer screening. *JAMA Netw Open*. 2024;7(12):e2451715.
doi:10.1001/jamanetworkopen.2024.51715

eAppendix. Model Description, Model Validation Results, and Sensitivity Analyses

eTable 1. Simulated Screening Histories Relative to Index Age

eTable 2. Test Characteristics and Complications Assumptions

eTable 3. Post-Colonoscopy Surveillance Assumptions

eTable 4. Societal Costs of Screening, Follow-Up, Surveillance Tests, and Complications (2020 USD)

eTable 5. Costs of Cancer Care (2020 USD)

eTable 6. Utility Losses of Screening, Follow-Up, Surveillance Tests, and Complications

eTable 7. Utility Losses of Cancer Care

eFigure 1. Sex- and Age-Specific CRC Incidence Rates for Cohorts With a Negative Colonoscopy 10 Years Prior and Cohorts With ≥1 Negative FIT Prior

eFigure 2. Sex- and Age-Specific CRC Incidence Rates by Number of Negative FIT Prior

eFigure 3. Sex- and Age-Specific CRC-Mortality Rates by Number of Negative FIT Prior

eFigure 4. Sex- and Age-Specific CRC Incidence Rates for Cohorts With ≥ 1 Negative FIT Prior and Cohorts With a Negative Colonoscopy + FIT (Hybrid)

eFigure 5. Sex- and Age-Specific CRC-Mortality Rates for Cohorts With ≥ 1 Negative FIT Prior and Cohorts With a Negative Colonoscopy + FIT (Hybrid)

eFigure 6. Comorbidity-Specific CRC Incidence Rates for Cohorts With a Negative Colonoscopy 10 Years Prior and Cohorts With ≥1 Negative FIT Prior

eFigure 7. Comorbidity-Specific CRC-Mortality Rates for Cohorts With a Negative Colonoscopy 10 Years Prior and Cohorts With ≥1 Negative FIT Prior

eFigure 8. Optimal CRC Screening Stopping Age by Modality for Screening Histories, Including No Screening or Only a Single Colonoscopy

eFigure 9. Optimal CRC Screening Stopping Age by Modality for Screening Histories, Only Including FITs

eFigure 10. Optimal CRC Screening Stopping Age by Modality for Screening Histories, Including a Single Colonoscopy 10 Years Prior and 0-5 FIT

eFigure 11. Optimal CRC Screening Stopping Age by Modality for Screening Histories, Including a Single Colonoscopy 15 Years Prior and 0-5 FIT

eFigure 12. Optimal CRC Screening Stopping Age by Modality for Screening Histories, Including a Single Colonoscopy 20 Years Prior and 0-5 FIT

eFigure 13. Optimal CRC Screening Stopping Age by Modality for Screening Histories, Including a Single Colonoscopy 25 Years Prior and 0-5 FIT

© 2024 Harlass M et al. *JAMA Network Open*

eFigure 14. Optimal CRC Screening Stopping Age by Modality for Screening Histories, Including a Single Colonoscopy 30 Years Prior and 0-5 FIT

eFigure 15. Estimated Clinical Outcomes of Continuing Annual FIT Screening for Cohorts From Age 76 Until the Estimated Stopping Age

eFigure 16. Costs per QALY Gained (QALYG) for Selected Strategies Stratified by Post-Colonoscopy Surveillance Assumptions

eReferences

This supplemental material has been provided by the authors to give readers additional information about their work.

© 2024 Harlass M et al. *JAMA Network Open*

eAppendix. Model Description, Model Validation Results, and Sensitivity Analyses

Model Description

The MISCAN models simulates individual life histories in a large cohort, representative of the US population in life expectancy and colorectal cancer risk. As simulated individuals age, they may develop one or multiple adenomas that can progress in size from small (≤ 5 mm) to medium (6-9 mm) to large (≥ 10 mm). Some adenomas develop into preclinical cancer (stages I to IV), and CRC can be clinically diagnosed at any stage due to the development of symptoms or through screening. Survival after clinical diagnosis depends on the stage at diagnosis, cancer location, and the individual's age. Screening alters some simulated life histories through cancer prevention (from detecting and removing adenomas) and early detection of cancers with more favorable survival. However, screening can also result in complications from screening or treatment procedures, over-diagnosis (i.e., detection of polyps and cancers that would not have led to the person's death before death from other causes), and over-treatment. The costs and effects of a screening strategy can be quantified by comparing the life histories of individuals with and without screening.

Model Validation Results

For the younger age groups (76-80 years), the observed incidence rates in the PRECISE sub-cohort were most consistent with simulated cohorts with five negative FITs in the previous five years (eFigure 1). The mortality rates were outside the confidence intervals for some simulated cohorts with less than three prior negative FITs (Figure 1). To evaluate the impact of prolonged prior screening (> 5 years prior), we simulated cohorts that underwent prolonged FIT testing (5-20 negative FIT) or hybrid screening with perfect adherence. Cohorts aged 76-80 years with prolonged prior FIT (eFigures 2-3) or hybrid screening (negative colonoscopy 10-30 years prior + 1-5 recent negative FIT, eFigures 4-5) showed incidence and mortality rates consistent with the observed risks from PRECISE. This is likely indicative of individuals from the PRECISE cohort having undergone prior testing not captured in the data, as those tests were outside the observation period.

Sensitivity Analyses

Our first sensitivity analysis assessed the impact of post-polypectomy surveillance on the cost-effectiveness outcomes and stopping ages. We assumed that individuals do not enter post-polypectomy surveillance after a positive screening or follow-up colonoscopy.

The second sensitivity analysis evaluated the potential impact of increasing colonoscopy complication rates with comorbidity status. To this end, we estimated relative risks for serious gastrointestinal, other gastrointestinal, and cardiovascular events, stratified by comorbid conditions, based on a study of complication rates in elderly Medicaid beneficiaries after outpatient colonoscopy.[1] The relative risks were calculated as the ratio of adjusted risk in individuals with a comorbid condition to adjusted risk in individuals without the condition. We mapped the relative risk for complications under each comorbid condition to a comorbidity status according to the same categorization used for the comorbidity-specific lifetables. If multiple comorbid conditions fell under the same comorbidity status, we calculated the average relative risk for that comorbidity status. Consequently, we mapped the average relative risk for individuals with chronic obstructive pulmonary disease and congestive heart failure to the severe comorbidity category, the relative risk for diabetics to the moderate category, and a relative risk of 1 to the category without comorbidities. As there was no data on risk for conditions included in the low comorbidity category, we used linear interpolation to impute a value. Finally, we re-scaled the relative risks so that the average relative risk of complications across comorbidity categories equaled 1 and multiplied the number of complications in each cohort by the comorbidity-specific weight.

eTable 1. Simulated Screening Histories Relative to Index Age

Negative colonoscopy	Negative FIT	Hybrid screening
10 y prior<br>15 y prior<br>20 y prior<br>25 y prior<br>30 y prior	5 recent FITs (1, 2, 3, 4, 5 y prior)<br>4 recent FITs (1, 2, 4, 5 y prior)<br>3 recent FITs (1, 3, 5 y prior)<br>2 recent FITs (1, 3 y prior)<br>1 recent FIT (1 y prior)	All combinations of FIT and colonoscopy columns
	3 FITs (3, 4, 5 y prior)<br>2 FITs (3, 5 y prior)<br>1 FIT (3 y prior)	

Abbreviations: FIT, fecal immunochemical test.

© 2024 Harlass M et al. *JAMA Network Open*

eTable 2. Test Characteristics and Complications Assumptions

**Test characteristics**		
	Screening modality	
	Colonoscopy	FIT
Sensitivity, %		
Small adenoma (≤ 5 mm)	41	0
Medium adenomas (6-9 mm)	85	11.4
Large adenomas (≥ 10 mm)	95	15.9
CRC, long before diagnosis	95	62.565
CRC, short before diagnosis	95	88.6
Specificity, %	86 (100)[a]	96.4
**Complication rates[b]**		
Fatal perforation[c]	0.0000142391	0
Serious gastrointestinal event[d]	Age-specific[g]	0
Other gastrointestinal event[e]	Age-specific[g]	0
Cardiovascular event[f]	Age-specific[g]	0

Abbreviations: CRC, colorectal cancer; FIT, fecal immunochemical test.

[a] MISCAN simulates a colonoscopy specificity of 1 (no false positives). The lack of specificity of colonoscopy is handled in post-processing

[b] applies only for positive colonoscopies

[c] Risk of dying from a colonoscopy at age 65 (Warren et al.[1], Gatto et al.[2], and Van Hees et al.[3]).

[d] Serious gastrointestinal events are perforations, gastrointestinal bleeding, or transfusions.

[e] Other gastrointestinal events are paralytic ileus, nausea, vomiting, dehydration, and abdominal pain.

[f] Cardiovascular events are myocardial infarction or angina, arrhythmias, congestive heart failure, cardiac or respiratory arrest, syncope, hypotension, or shock.

[g] Formulas for age-specific excess risks:

Serious GI events: $1 / [\exp(9.27953 - 0.06105 \times Age) + 1] - 1 / [\exp(10.78719 - 0.06105 \times Age) + 1]$

Other GI events: $1 / [\exp(8.81404 - 0.05903 \times Age) + 1] - 1 / [\exp(9.61197 - 0.05903 \times Age) + 1]$

Cardiovascular events: $1 / [\exp(9.09053 - 0.07056 \times Age) + 1] - 1 / [\exp(9.38297 - 0.07056 \times Age) +1]$

© 2024 Harlass M et al. *JAMA Network Open*

eTable 3. Post-Colonoscopy Surveillance Assumptions

Finding at second-most recent colonoscopy[a]	Finding at first-most recent colonoscopy[a]	Interval[b] to next colonoscopy, y
No prior colonoscopy	Normal colonoscopy[c]	See note below[d]
	1-2 adenomas <10 mm	7
	3-4 adenomas <10 mm	3
	10 adenomas <10 mm or any adenoma ≥10 mm	3
	> 10 adenomas	1
Normal colonoscopy[c]	Normal colonoscopy[c]	10
	1-2 adenomas <10 mm	7
	3-4 adenomas <10 mm	3
	5-10 adenomas <10 mm or any adenoma ≥10 mm	3
	> 10 adenomas	1
1-2 adenomas <10 mm	Normal colonoscopy[c]	10
	1-2 adenomas <10 mm	7
	3-4 adenomas <10 mm	3
	5-10 adenomas <10 mm or any adenoma ≥10 mm	3
	> 10 adenomas	1
3-4 adenomas <10 mm	Normal colonoscopy[c]	10
	1-2 adenomas <10 mm	7
	3-4 adenomas <10 mm	3
	5-10 adenomas <10 mm or any adenoma ≥10 mm	3
	> 10 adenomas	1
5-10 adenomas <10 mm or any adenoma ≥10 mm	Normal colonoscopy[c]	5
	1-2 adenomas <10 mm	5
	3-4 adenomas <10 mm	3
	5-10 adenomas <10 mm or any adenoma ≥10 mm	3
	> 10 adenomas	1
> 10 adenomas of any size	Normal colonoscopy[c]	5
	1-2 adenomas <10 mm	5
	3-4 adenomas <10 mm	3
	5-10 adenomas <10 mm or any adenoma ≥10 mm	3
	>10 adenomas	1

[a] This table omits the case where CRC is detected at a screening, diagnostic, or surveillance colonoscopy because the CISNET CRC models do not simulate detailed events following CRC diagnosis.

[b] The Multi-Society Task Force provides a range for some intervals (e.g., the interval for 3-4 adenomas <10 mm is 3-5 years). In such cases, we selected the shortest intervals provided.

[c] normal colonoscopy = no adenomas, SSPs (currently not simulated), or CRC is detected.

[d] A person whose first screening or diagnostic colonoscopy is normal does not enter surveillance but resumes screening 10 years after the normal colonoscopy.

© 2024 Harlass M et al. *JAMA Network Open*

eTable 4. Societal Costs of Screening, Follow-Up, Surveillance Tests, and Complications (2020 USD)

**Test costs**				
	Test costs ($)	Patient time (h)	Time costs ($)[a]	Total costs (societal, 2020 $)
Colonoscopy[b]				
Screening colonoscopy without lesion removal	891.37	39.93	805.39	1,696.76
Follow-up colonoscopy without lesion removal	891.01	39.93	805.39	1,696.40
Surveillance colonoscopy without lesion removal	890.65	39.93	805.39	1,696.04
Any colonoscopy with lesion removal	1,328.22	39.93	805.39	2,133.61
FIT[c]	23.82	1	20.17	43.99
**Complications**				
	Total complication cost (societal, 2020 $)			
Serious gastrointestinal event	13,573			
Other gastrointestinal event	8,574			
Cardiovascular event	10,985			

Abbreviations: FIT, fecal immunochemical test.
[a] We assumed an hour of patient time to equal the median wage rate in 2020 ($20.17) according to data from the US Bureau of Labor Statistics (BLS))
[b] Colonoscopy costs were based on an analysis of 2020 Centers for Medicare and Medicaid Services (CMS) data
[c] FIT costs were based on 2017 Clinical Laboratory Fee Schedule data, inflated to 2020 USD using 2020 CPI data for All Urban Consumers (CPI-U)

© 2024 Harlass M et al. *JAMA Network Open*

eTable 5. Costs of Cancer Care (2020 USD)

**Phase of cancer care**	**Annual societal costs (2020 $)**[a]			
	Stage I	Stage II	Stage III	Stage IV
Initial phase	46,681	64,183	91,071	132,991
Continuing phase	4,695	5,397	8,139	37,134
Terminal phase, death CRC	90,080	100,829	105,300	130,973
Terminal phase, death other causes	27,782	29,471	38,235	84,111

Abbreviations: CRC, colorectal cancer
[a] from Peterse et al.[4], inflated to 2020 USD using 2020 CPI data for All Urban Consumers (CPI-U)

© 2024 Harlass M et al. *JAMA Network Open*

eTable 6. Utility Losses of Screening, Follow-Up, Surveillance Tests, and Complications

**Utility loss for test procedure itself[a]**			
	Disutility	Time disutility is applied (h)	Utility loss per event
Colonoscopy	0.12	36.22	0.000496
FIT	0	1	0
**Utility loss for waiting for test results[a]**			
	Disutility	Time disutility is applied (h)	Utility loss per event
without lesion removal	0	0	0
Colonoscopy with lesion removal	0.033036	10	0.000905
FIT	0.003304	7	0.000063
**Utility loss for waiting for diagnostic follow-up colonoscopy[a]**			
	Disutility	Time disutility is applied (h)	Utility loss per event
FIT	0.033036	14	0.001267
**Total utility loss per test[a]**			
	Disutility negative test	Disutility positive test	
Colonoscopy	0.000496	0.001401	
FIT	0.000063	0.001330	
**Utility loss for complications[a]**			
	Disutility	Time disutility is applied (h)	Utility loss per event
Serious gastrointestinal event	0.5	96	0.0055
Other gastrointestinal event	0.5	48	0.0027
Cardiovascular event	0.5	84	0.0048

Abbreviations: fecal immunochemical test.
[a] from Peterse et al.[4]

© 2024 Harlass M et al. *JAMA Network Open*

eTable 7. Utility Losses of Cancer Care

Phase of cancer care	Utility loss[a]			
	Stage I	Stage II	Stage III	Stage IV
Initial phase	0.12	0.18	0.24	0.70
Continuing phase	0.05	0.05	0.24	0.70
Terminal phase, death CRC	0.70	0.70	0.70	0.70
Terminal phase, death other causes	0.05	0.05	0.24	0.70

[a] from Peterse et al.[4]

© 2024 Harlass M et al. *JAMA Network Open*

eFigure 1. Sex- and Age-Specific CRC Incidence Rates for Cohorts With a Negative Colonoscopy 10 Years Prior and Cohorts With ≥1 Negative FIT Prior

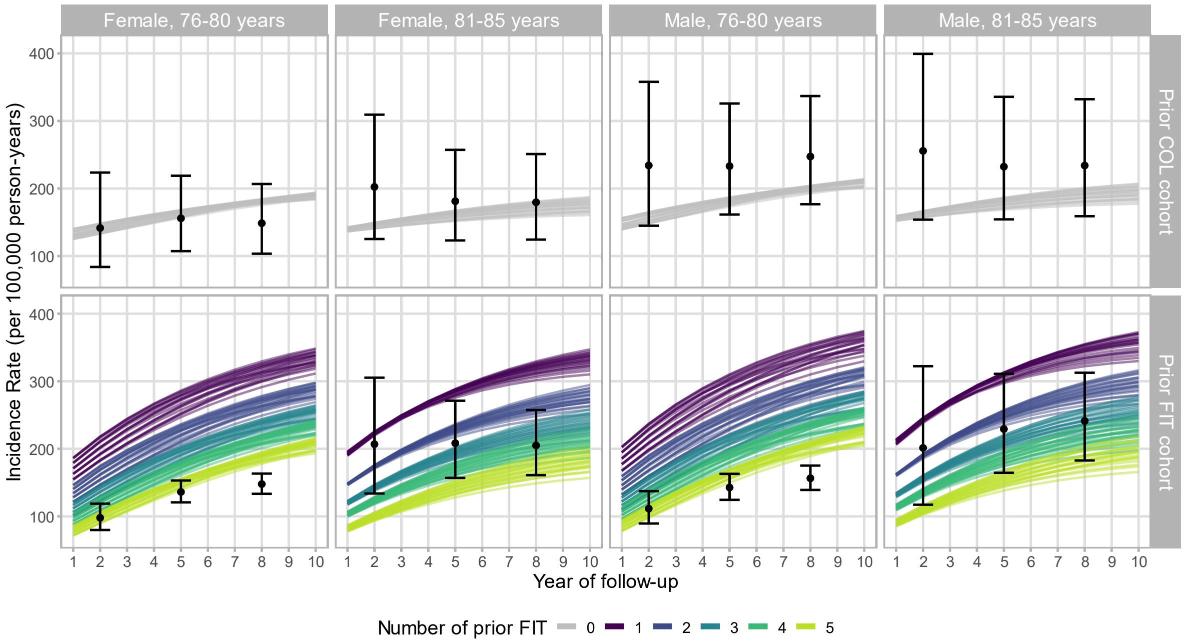

Abbreviations: COL, colonoscopy; FIT, fecal immunochemical test.
The points and bars indicate the observed incidence in the PRECISE cohort and 95% CIs for patients with a negative colonoscopy 10y prior or a negative FIT 1 year prior, respectively. The lines represent the simulated incidence for each index age (76-85y) over 10 years of follow-up (x-axis), stratified by number of FITs over the last 5 years (colors).

eFigure 2. Sex- and Age-Specific CRC Incidence Rates by Number of Negative FIT Prior

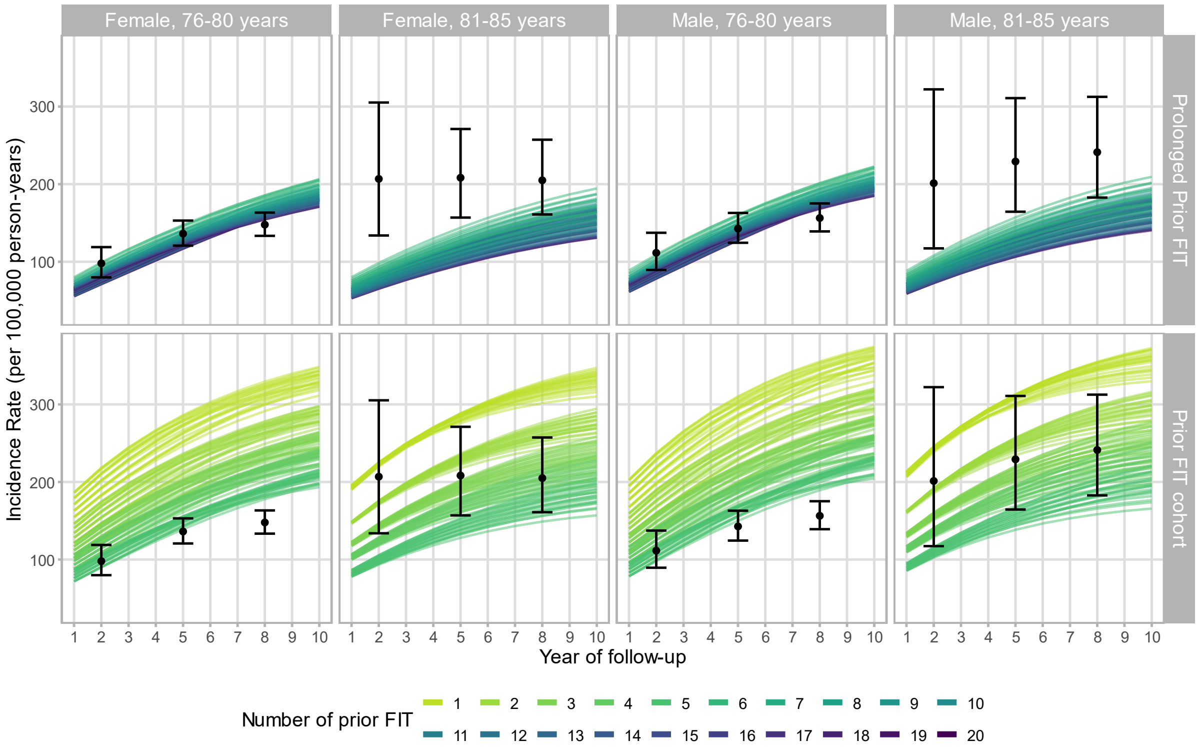

Abbreviations: COL, colonoscopy; FIT, fecal immunochemical test.
The points and bars indicate the observed incidence in the PRECISE cohort and 95% CIs for patients with 1-5 negative FIT in the 5 years prior. The lines represent the simulated incidence for each index age (76-85y) over 10 years of follow-up (x-axis), stratified by number of negative FITs (colors).

eFigure 3. Sex- and Age-Specific CRC-Mortality Rates by Number of Negative FIT Prior

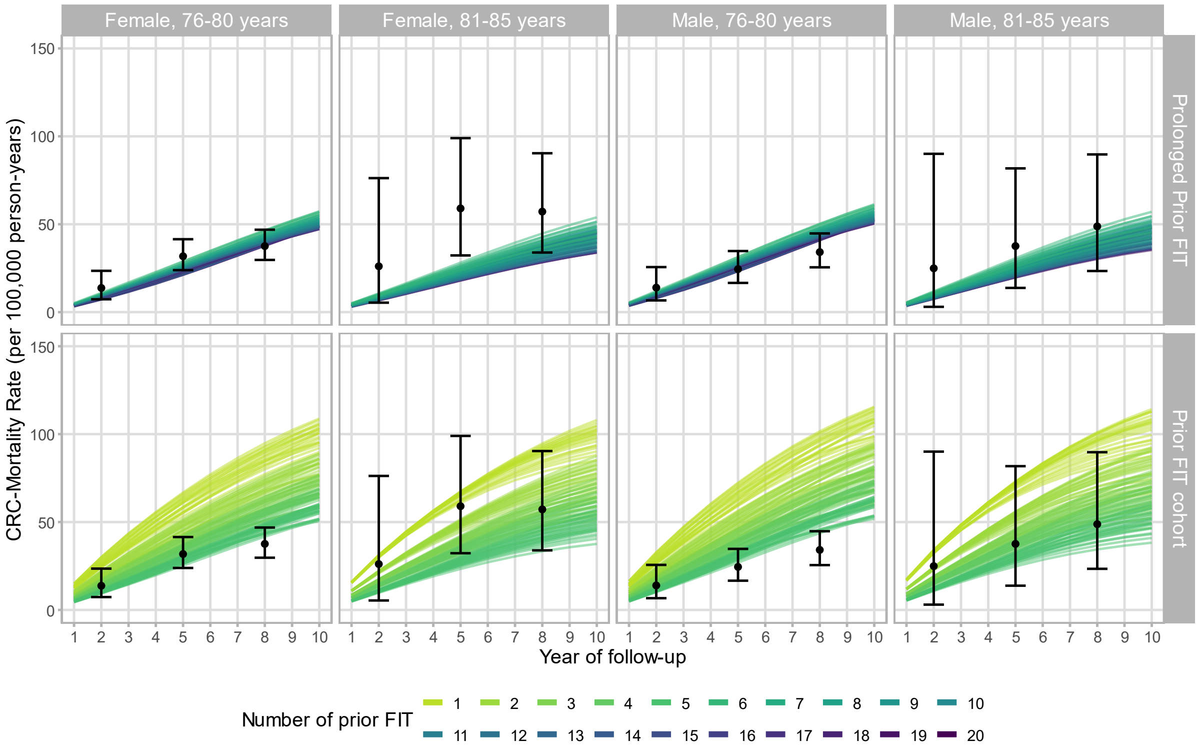

Abbreviations: COL, colonoscopy; FIT, fecal immunochemical test.
The points and bars indicate the observed mortality in the PRECISE cohort and 95% CIs for patients with 1-5 negative FIT in the 5 years prior. The lines represent the simulated mortality for each index age (76-85y) over 10 years of follow-up (x-axis), stratified by number of negative FITs (colors).

© 2024 Harlass M et al. *JAMA Network Open*

eFigure 4. Sex- and Age-Specific CRC Incidence Rates for Cohorts With ≥ 1 Negative FIT Prior and Cohorts With a Negative Colonoscopy + FIT (Hybrid)

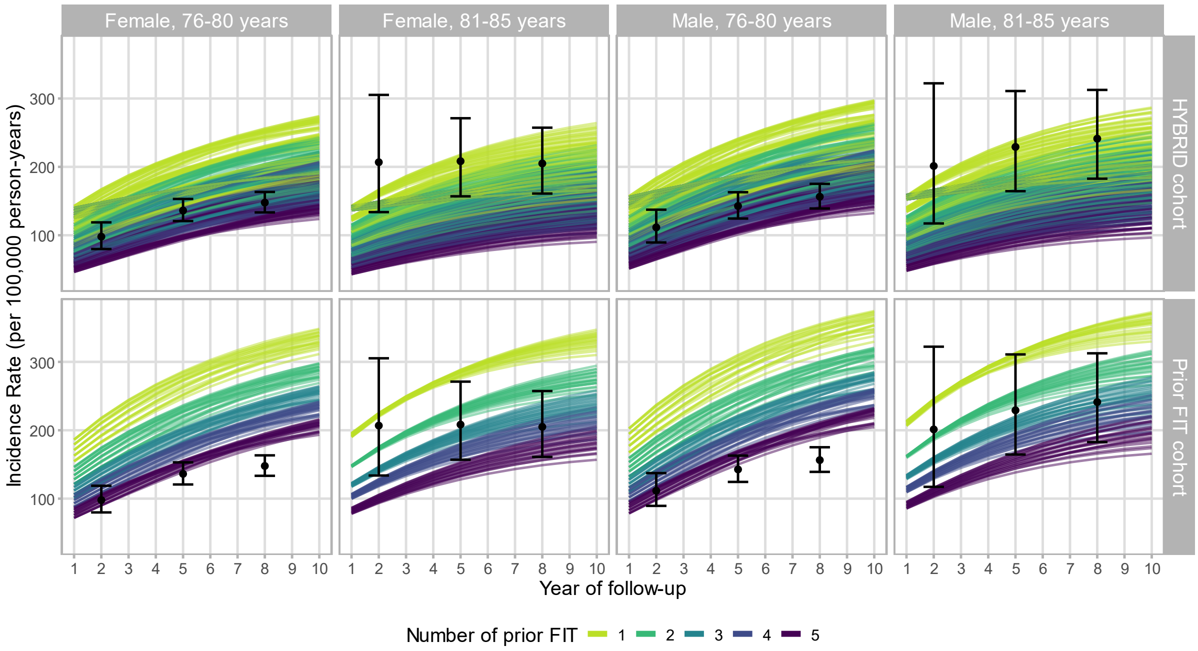

Abbreviations: COL, colonoscopy; FIT, fecal immunochemical test.
The points and bars indicate the observed incidence in the PRECISE cohort and 95% CIs for patients with a negative FIT 1 year prior. The lines represent the simulated incidence for each index age (76-85y) over 10 years of follow-up (x-axis), stratified by number of FITs over the last 5 years (colors).

eFigure 5. Sex- and Age-Specific CRC-Mortality Rates for Cohorts With ≥ 1 Negative FIT Prior and Cohorts With a Negative Colonoscopy + FIT (Hybrid)

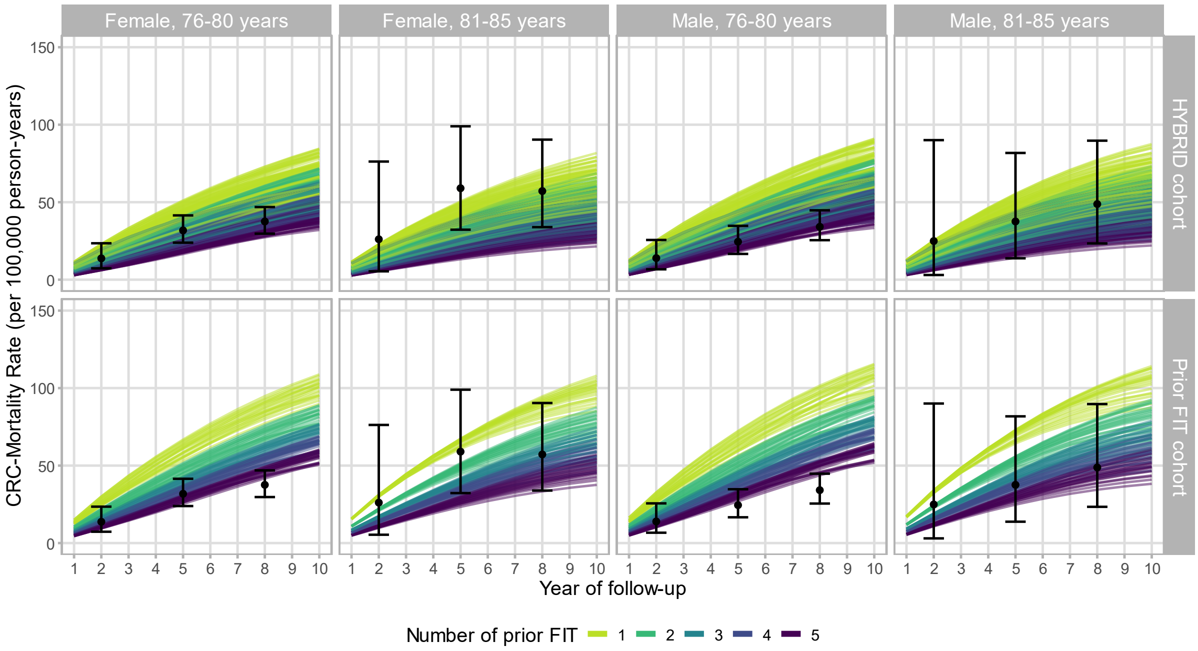

Abbreviations: COL, colonoscopy; FIT, fecal immunochemical test.
The points and bars indicate the observed mortality in the PRECISE cohort and 95% CIs for patients with a negative FIT 1 year prior. The lines represent the simulated mortality for each index age (76-85y) over 10 years of follow-up (x-axis), stratified by number of FITs over the last 5 years (colors).

© 2024 Harlass M et al. *JAMA Network Open*

eFigure 6. Comorbidity-Specific CRC Incidence Rates for Cohorts With a Negative Colonoscopy 10 Years Prior and Cohorts With ≥1 Negative FIT Prior

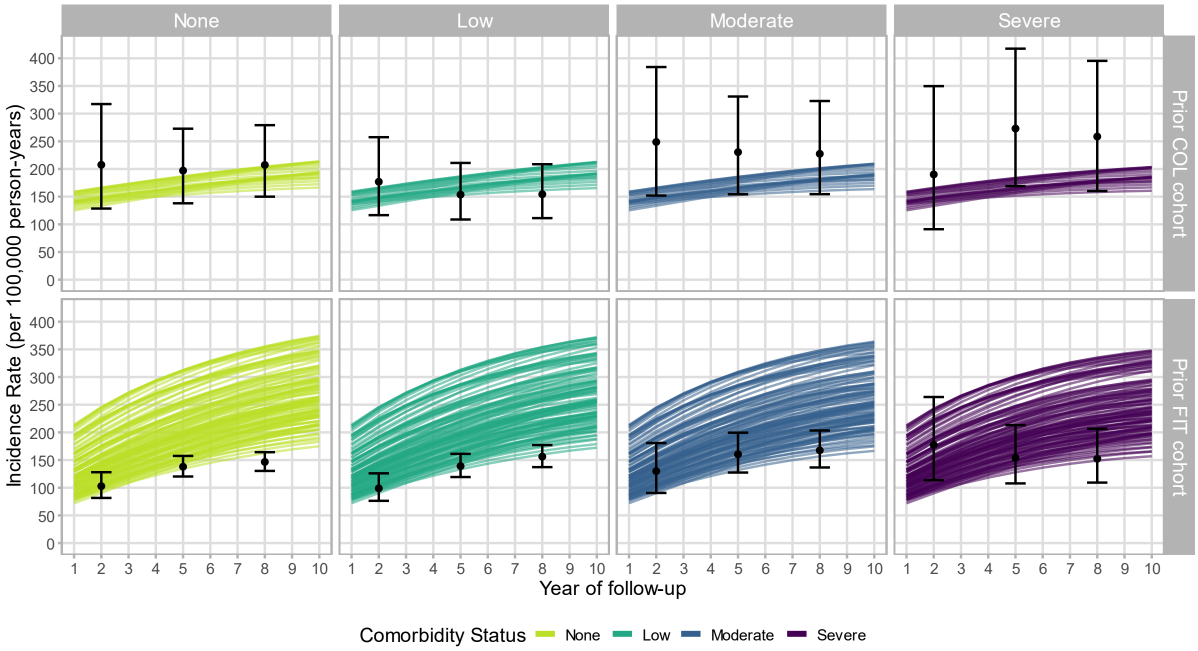

Abbreviations: COL, colonoscopy; FIT, fecal immunochemical test.
The points and bars indicate the observed incidence in the PRECISE cohort and 95% CIs for patients with a negative colonoscopy 10y prior or a negative FIT 1 year prior, respectively. The lines represent the simulated incidence for each index age (76-85y) over 10 years of follow-up (x-axis), stratified by comorbidity status (colors).

© 2024 Harlass M et al. *JAMA Network Open*

eFigure 7. Comorbidity-Specific CRC-Mortality Rates for Cohorts With a Negative Colonoscopy 10 Years Prior and Cohorts With ≥1 Negative FIT Prior

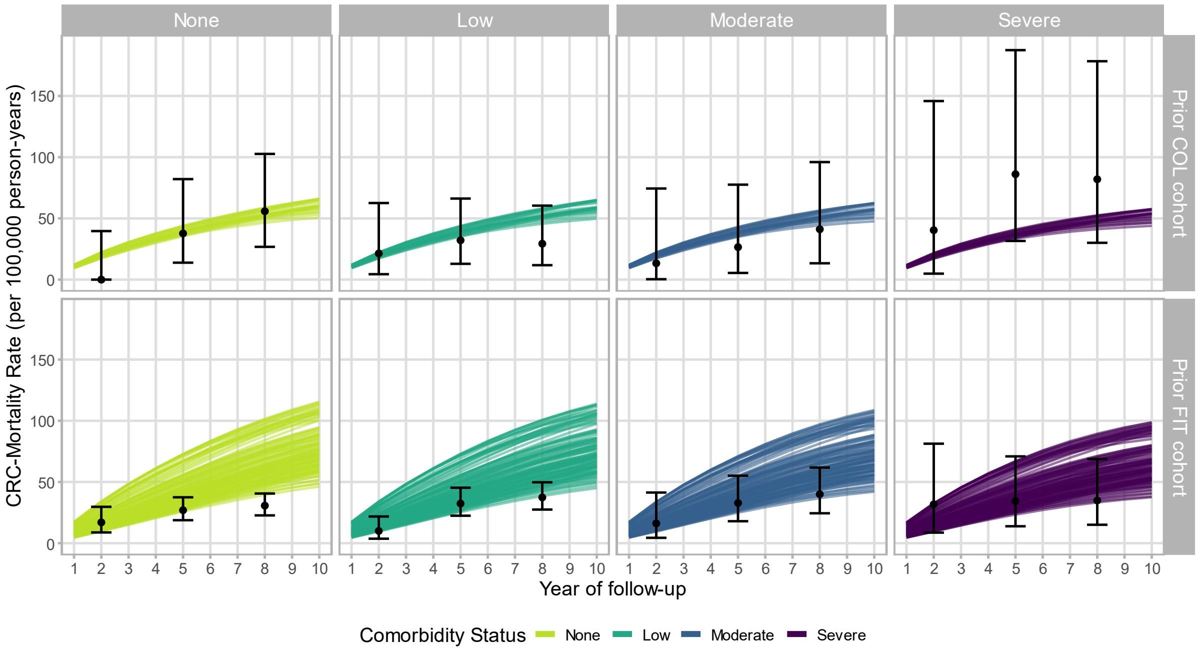

Abbreviations: COL, colonoscopy; FIT, fecal immunochemical test.
The points and bars indicate the observed mortality in the PRECISE cohort and 95% CIs for patients with a negative colonoscopy 10y prior or a negative FIT 1 year prior, respectively. The lines represent the simulated mortality for each index age (76-85y) over 10 years of follow-up (x-axis), stratified by comorbidity status (colors).

© 2024 Harlass M et al. *JAMA Network Open*

eFigure 8. Optimal CRC Screening Stopping Age by Modality for Screening Histories, Including No Screening or Only a Single Colonoscopy

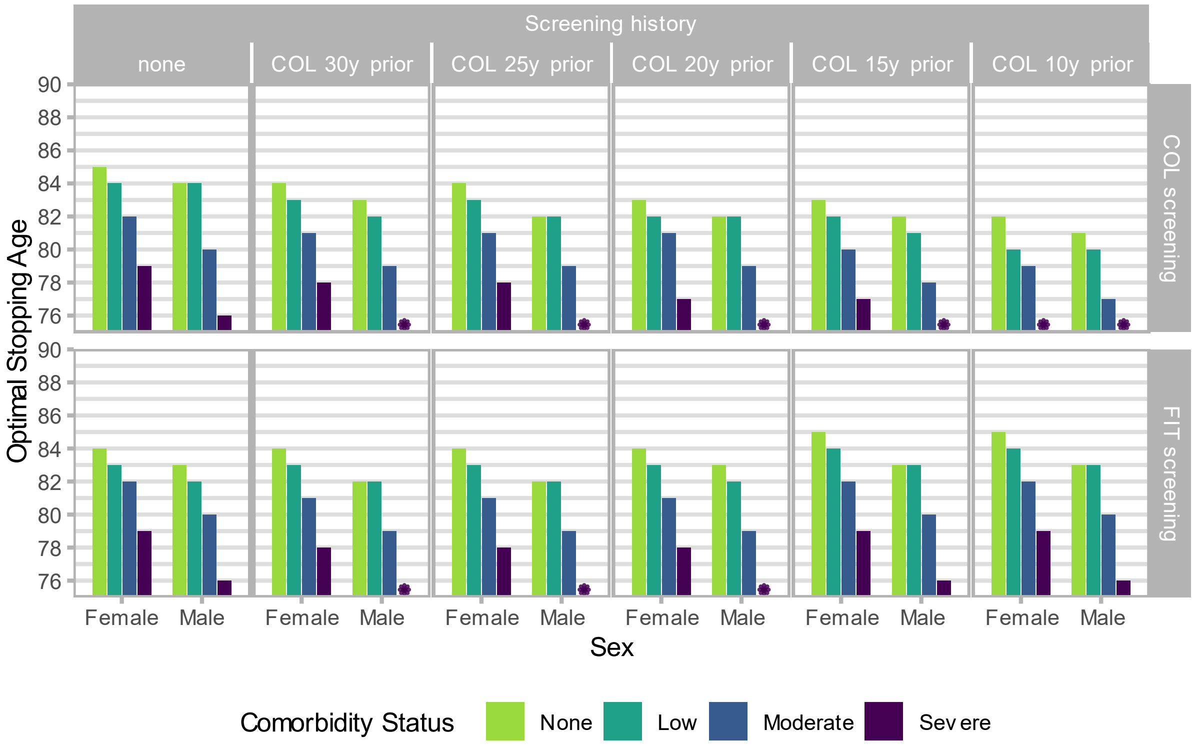

Abbreviations: COL, colonoscopy; FIT, fecal immunochemical test
Optimal stopping age was defined as the oldest age at which one more screening is still cost-effective.
* screening after age 75 is not cost-effective.

© 2024 Harlass M et al. *JAMA Network Open*

eFigure 9. Optimal CRC Screening Stopping Age by Modality for Screening Histories Only, Including FITs

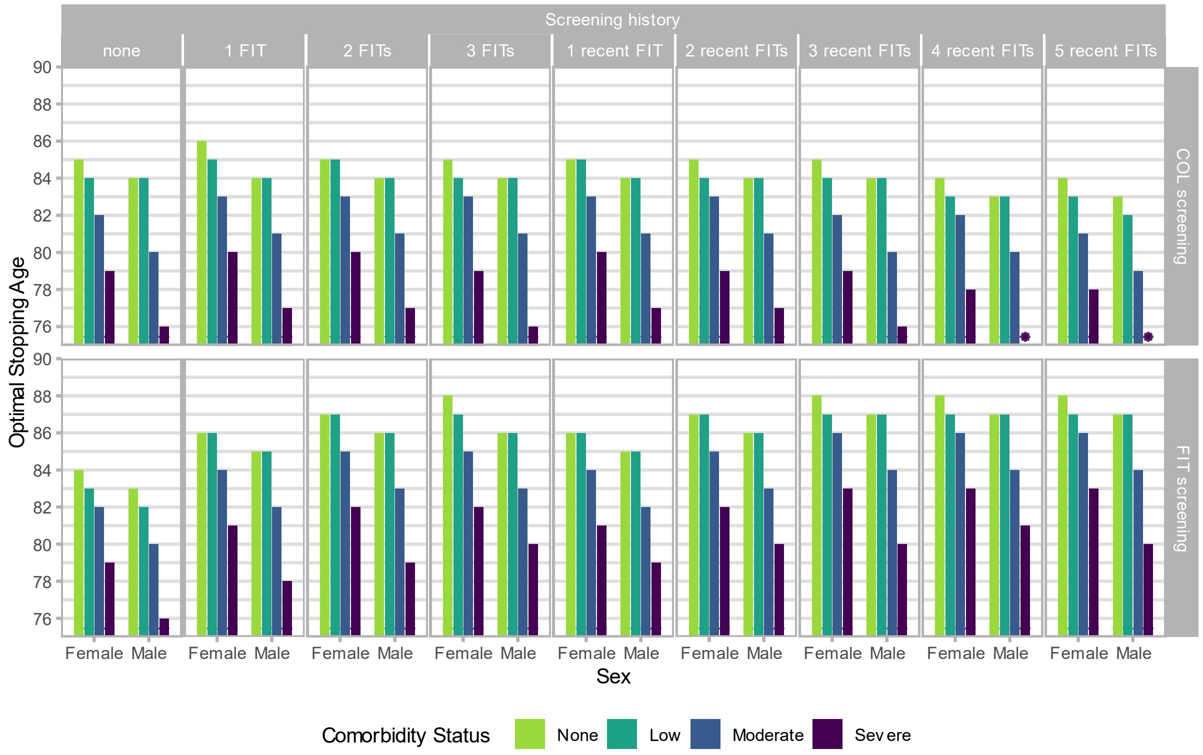

Abbreviations: COL, colonoscopy; FIT, fecal immunochemical test
Optimal stopping age was defined as the oldest age at which one more screening is still cost-effective.
* screening after age 75 is not cost-effective.

© 2024 Harlass M et al. *JAMA Network Open*

eFigure 10. Optimal CRC Screening Stopping Age by Modality for Screening Histories, Including a Single Colonoscopy 10 Years Prior and 0-5 FIT

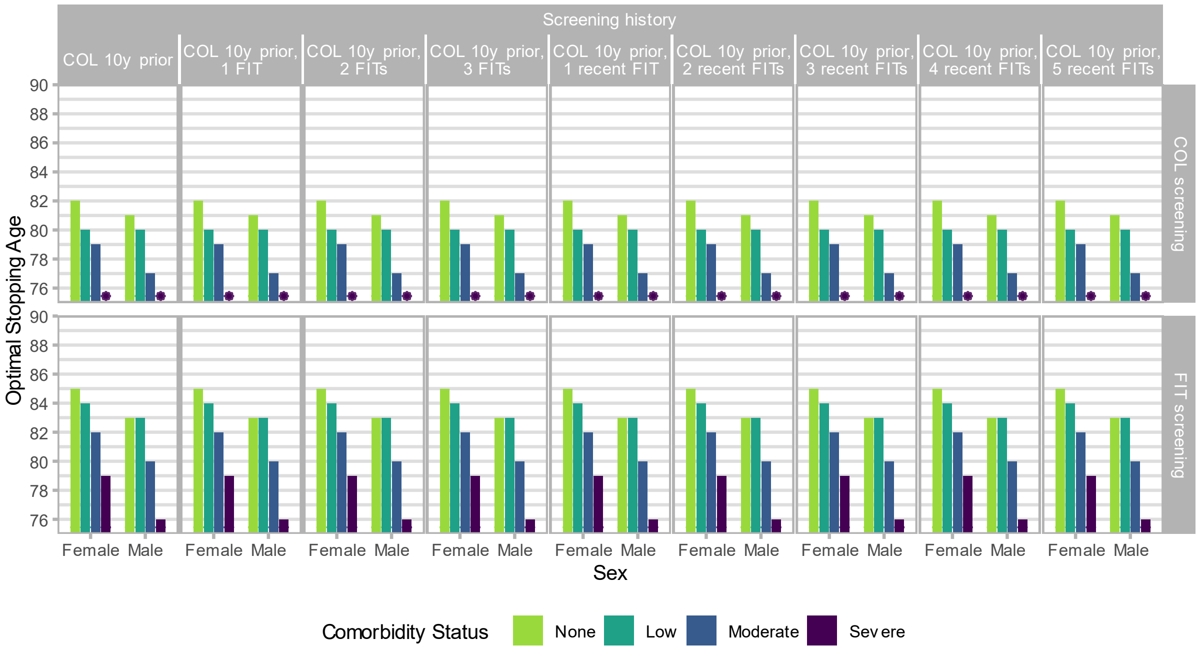

Abbreviations: COL, colonoscopy; FIT, fecal immunochemical test
Optimal stopping age was defined as the oldest age at which one more screening is still cost-effective.
* screening after age 75 is not cost-effective.

© 2024 Harlass M et al. *JAMA Network Open*

eFigure 11. Optimal CRC Screening Stopping Age by Modality for Screening Histories, Including a Single Colonoscopy 15 Years Prior and 0-5 FIT

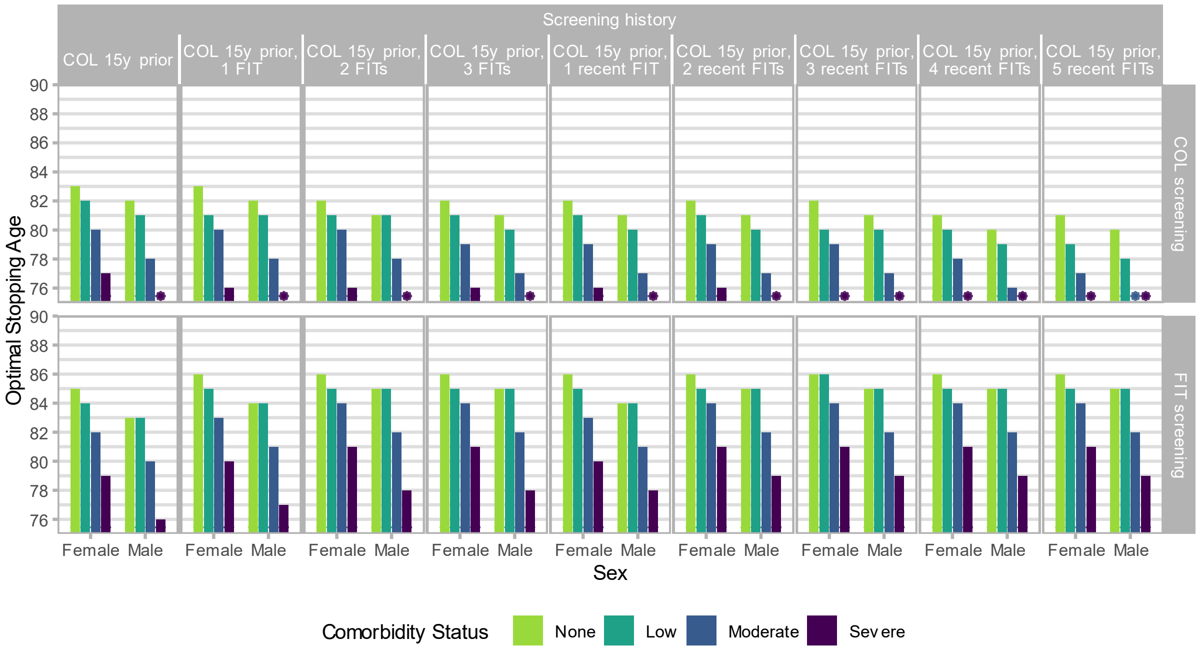

Abbreviations: COL, colonoscopy; FIT, fecal immunochemical test
Optimal stopping age was defined as the oldest age at which one more screening is still cost-effective.* screening after age 75 is not cost-effective.

© 2024 Harlass M et al. *JAMA Network Open*

eFigure 12. Optimal CRC Screening Stopping Age by Modality for Screening Histories, Including a Single Colonoscopy 20 Years Prior and 0-5 FIT

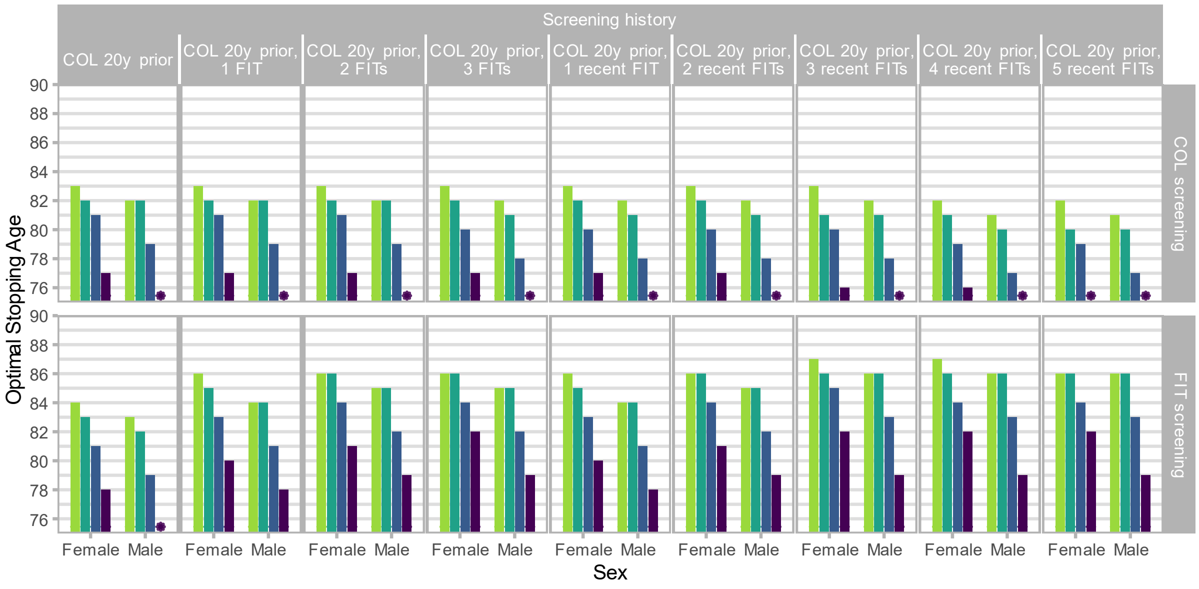

Abbreviations: COL, colonoscopy; FIT, fecal immunochemical test
Optimal stopping age was defined as the oldest age at which one more screening is still cost-effective.
* screening after age 75 is not cost-effective.

© 2024 Harlass M et al. *JAMA Network Open*

eFigure 13. Optimal CRC Screening Stopping Age by Modality for Screening Histories, Including a Single Colonoscopy 25 Years Prior and 0-5 FIT

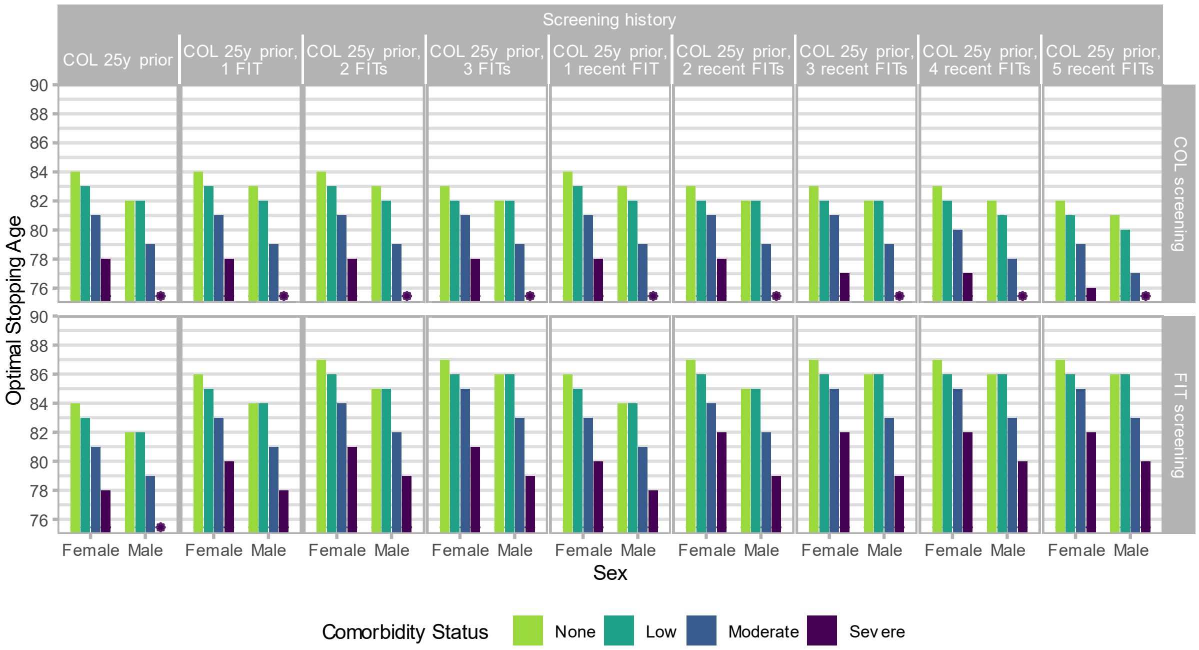

Abbreviations: COL, colonoscopy; FIT, fecal immunochemical test
Optimal stopping age was defined as the oldest age at which one more screening is still cost-effective.
* screening after age 75 is not cost-effective.

© 2024 Harlass M et al. *JAMA Network Open*

eFigure 14. Optimal CRC Screening Stopping Age by Modality for Screening Histories, Including a Single Colonoscopy 30 Years Prior and 0-5 FIT

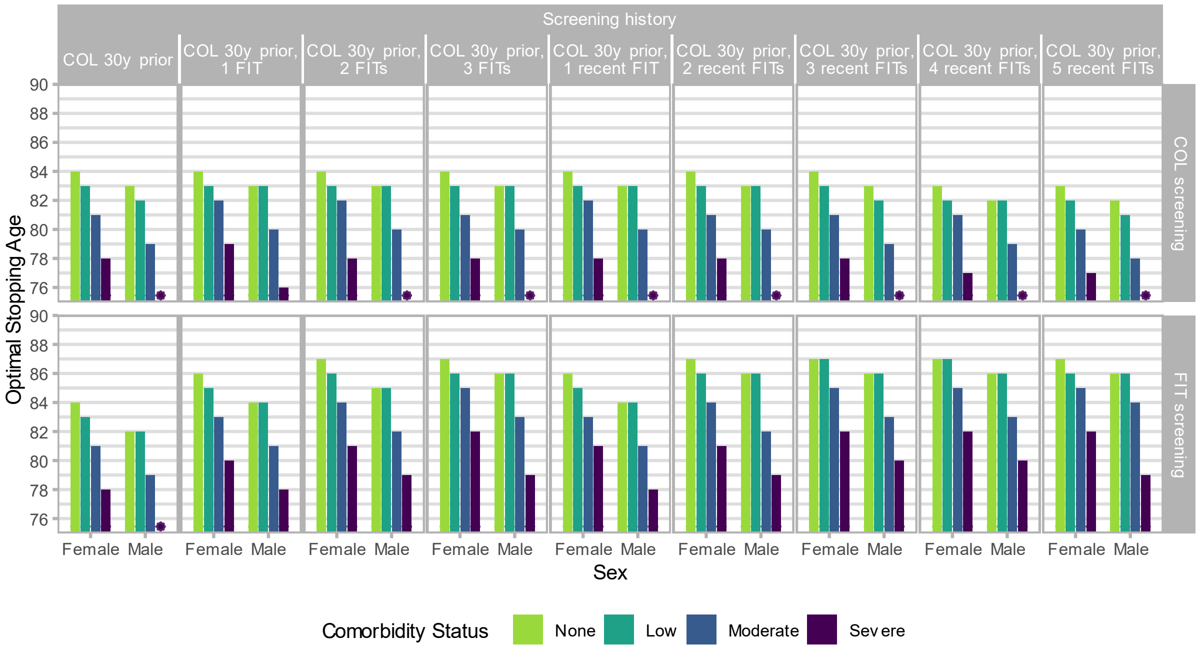

Abbreviations: COL, colonoscopy; FIT, fecal immunochemical test
Optimal stopping age was defined as the oldest age at which one more screening is still cost-effective.
* screening after age 75 is not cost-effective.

© 2024 Harlass M et al. *JAMA Network Open*

eFigure 15. Estimated Clinical Outcomes of Continuing Annual FIT Screening for Cohorts From Age 76 Until the Estimated Stopping Age

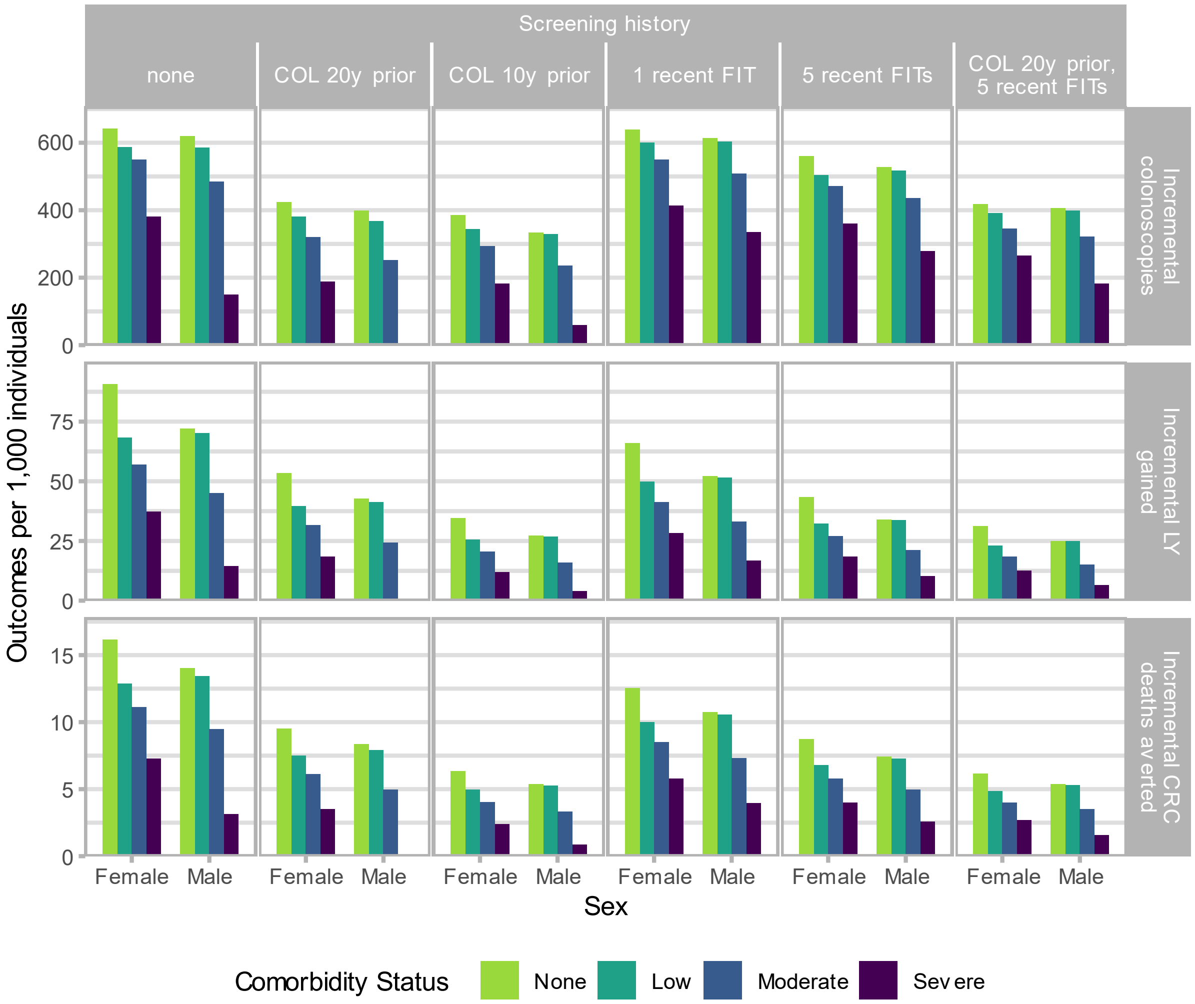

Abbreviations: COL, colonoscopy; FIT, fecal immunochemical test; CRC, colorectal cancer.
As colonoscopy screening was not cost-effective after age 86, the outcomes of colonoscopy screening from age 76 until the estimated stopping age match the values reported in eTable 8.

© 2024 Harlass M et al. *JAMA Network Open*

eFigure 16. *Costs per QALY Gained (QALYG) for Selected Strategies Stratified Post-Colonoscopy Surveillance Assumptions*

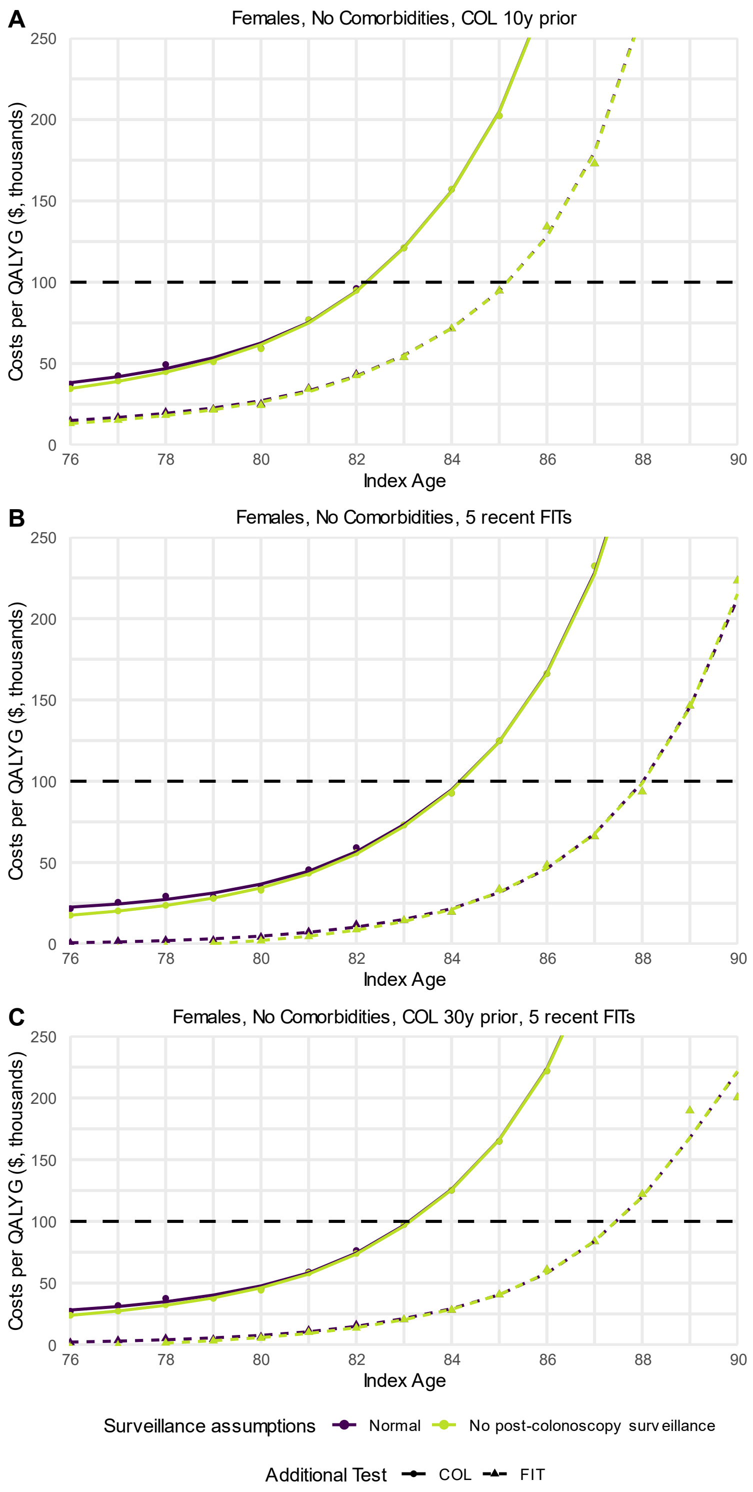

Abbreviations: COL, colonoscopy; FIT, fecal immunochemical test

The dots and triangles represent the estimated cost per QALYG with one additional colonoscopy and FIT. The colored lines indicated the smoothed ICERs calculated from smoothed costs and QALYGs. The black horizontal line represents the willingness-to-pay threshold of $100,000/QALYG.

© 2024 Harlass M et al. *JAMA Network Open*

eReferences

1. Warren JL, Klabunde CN, Mariotto AB, Meekins A, Topor M, Brown ML, et al. Adverse events after outpatient colonoscopy in the Medicare population. Ann Intern Med. 2009;150(12):849-57, W152.
2. Gatto NM, Frucht H, Sundararajan V, Jacobson JS, Grann VR, Neugut AI. Risk of perforation after colonoscopy and sigmoidoscopy: a population-based study. J Natl Cancer Inst. 2003;95(3):230-6.
3. van Hees F, Zauber AG, Klabunde CN, Goede SL, Lansdorp-Vogelaar I, van Ballegooijen M. The appropriateness of more intensive colonoscopy screening than recommended in Medicare beneficiaries: a modeling study. JAMA Intern Med. 2014;174(10):1568-76.
4. Peterse EFP, Meester RGS, de Jonge L, Omidvari AH, Alarid-Escudero F, Knudsen AB, et al. Comparing the Cost-Effectiveness of Innovative Colorectal Cancer Screening Tests. J Natl Cancer Inst. 2021;113(2):154-61.

© 2024 Harlass M et al. *JAMA Network Open*
