# Supplementary material for: Optimal Stopping Ages for Colorectal Cancer Screening
Source: JAMA Netw Open. 2024 Dec 19;7(12):e2451715. doi: 10.1001/jamanetworkopen.2024.51715 (PMC11659906; doi:10.1001/jamanetworkopen.2024.51715)
Supplement: Supplement 3. — Data Sharing Statement [file jamanetwopen-e2451715-s003.pdf]

Data Sharing Statement

Harlass. Optimal Stopping Ages for Colorectal Cancer Screening. *JAMA Netw Open*.
Published December 19, 2024. doi:10.1001/jamanetworkopen.2024.51715

Data
Data available: Yes
Data types: Data (not involving human participants)
How to access data: The simulated data supporting the results of this study are available from the corresponding author upon request (m.harlass@erasmusmc.nl). Interested researchers can contact the corresponding author for more insight into the MISCAN model that provided the simulated data.
When available: With publication

Supporting Documents
Document types: None

Additional Information
Who can access the data: Anyone requesting the data
Types of analyses: Any purpose
Mechanisms of data availability: Without investigator support
